# Pan-cancer spatially resolved single-cell analysis reveals the crosstalk between cancer-associated fibroblasts and tumor microenvironment

**DOI:** 10.1186/s12943-023-01876-x

**Published:** 2023-10-13

**Authors:** Chenxi Ma, Chengzhe Yang, Ai Peng, Tianyong Sun, Xiaoli Ji, Jun Mi, Li Wei, Song Shen, Qiang Feng

**Affiliations:** 1 Department of Human Microbiome and Periodontology and Implantology and Orthodontics, School and Hospital of Stomatology, Cheeloo College of Medicine, Shandong University and Shandong Key Laboratory of Oral Tissue Regeneration and Shandong Engineering Laboratory for Dental Materials and Oral Tissue Regeneration and Shandong Provincial Clinical Research Center for Oral Diseases, Jinan, 250012 China; 2 Department of Oral and Maxillofacial Surgery, Qilu Hospital of Shandong University, Jinan, Shandong China; 3 Institute of Stomatology, Shandong University, Jinan, Shandong China; 4 Department of Stomatology, Central Hospital Affiliated to Shandong First Medical University, No.105 Jiefang Road, Jinan, Shandong China; 5 State Key Laboratory of Microbial Technology, Shandong University, Qingdao, 266237 China

**Keywords:** Spatial transcriptomics, Single-cell RNA sequencing, Pan-cancer analysis, Cancer-associated fibroblasts, Tumor microenvironment, Tumor immunotherapy

## Abstract

Cancer-associated fibroblasts (CAFs) are a heterogeneous cell population that plays a crucial role in remodeling the tumor microenvironment (TME). Here, through the integrated analysis of spatial and single-cell transcriptomics data across six common cancer types, we identified four distinct functional subgroups of CAFs and described their spatial distribution characteristics. Additionally, the analysis of single-cell RNA sequencing (scRNA-seq) data from three additional common cancer types and two newly generated scRNA-seq datasets of rare cancer types, namely epithelial-myoepithelial carcinoma (EMC) and mucoepidermoid carcinoma (MEC), expanded our understanding of CAF heterogeneity. Cell–cell interaction analysis conducted within the spatial context highlighted the pivotal roles of matrix CAFs (mCAFs) in tumor angiogenesis and inflammatory CAFs (iCAFs) in shaping the immunosuppressive microenvironment. In patients with breast cancer (BRCA) undergoing anti-PD-1 immunotherapy, iCAFs demonstrated heightened capacity in facilitating cancer cell proliferation, promoting epithelial-mesenchymal transition (EMT), and contributing to the establishment of an immunosuppressive microenvironment. Furthermore, a scoring system based on iCAFs showed a significant correlation with immune therapy response in melanoma patients. Lastly, we provided a web interface (https://chenxisd.shinyapps.io/pancaf/) for the research community to investigate CAFs in the context of pan-cancer.

**Supplementary Information:**

The online version contains supplementary material available at 10.1186/s12943-023-01876-x.

## Introduction

Tumors display extensive heterogeneity, with cancer cells engaging in reciprocal interactions with their microenvironment, forming a complex ecosystem [1]. Cancer-associated fibroblasts (CAFs), as one of the most prominent and abundant cell populations in the tumor microenvironment (TME) [2], have garnered significant attention in recent years. CAF's intricate interactions with stromal components and immune cells play a crucial role in orchestrating TME reorganization, encompassing processes such as angiogenesis, extracellular matrix (ECM) remodeling, and immune evasion [3–5]. At present, the crucial role of CAFs has been largely overlooked by most therapies, including immunotherapy and chemotherapy [3]. Our current understanding of the interplay between CAFs and components of TME is insufficient to support the development of reliable treatment strategies. Further research is needed to deepen our understanding of these interactions and pave the way for effective therapeutic interventions.

In recent years, the application of single-cell transcriptomics has unraveled the heterogeneity of CAFs within many cancer types, such as bladder carcinoma (BC) [6], head and neck squamous cell carcinoma (HNSCC) [7], papillary thyroid carcinoma (PTC) [8], and lung cancer (LC) [9]. Furthermore, two recent unbiased studies based on single-cell RNA sequencing (scRNA-seq) explored the heterogeneity and plasticity of CAFs from a pan-cancer perspective and revealed the conservation of CAF phenotypes across cancer types [10, 11]. Although scRNA-seq provides an unprecedented opportunity to systematically dissect the heterogeneity of CAFs, the loss of spatial information during tissue dissociation hinders the study of the crosstalk between CAFs and TME. Recently developed spatial transcriptomics (ST) can obtain whole-transcriptome data within tissue sections, thereby preserving the spatial position information of cells [12]. Therefore, orthogonal integration of scRNA-seq data and ST data will help determine the spatial distribution characteristics of CAFs and further dissect the cellular communication between CAFs and TME.

In this study, we have delineated the landscape of CAFs in six common cancer types and described the unique functional features of these subtypes. We also analyzed scRNA-seq data of three additional common tumors and two newly sequenced rare tumors to expand our understanding of CAF heterogeneity. A spatial single-cell transcriptomic atlas spanning six tumors, including 744,289 cells, generated by integrating scRNA-seq data and ST data was used to describe the spatial distribution characteristics of CAFs and to characterize the complex interactions between CAFs and TME. Notably, a score generated based on inflammatory CAFs (iCAFs) showed a significant correlation with the response of melanoma patients to immunotherapy. In summary, our integrated data resources provide novel insights and guidance for the development of therapeutic strategies targeting CAFs in TME.

## Results

### Construction of a pan-cancer spatial single-cell transcriptome atlas

**Fig. 1:**
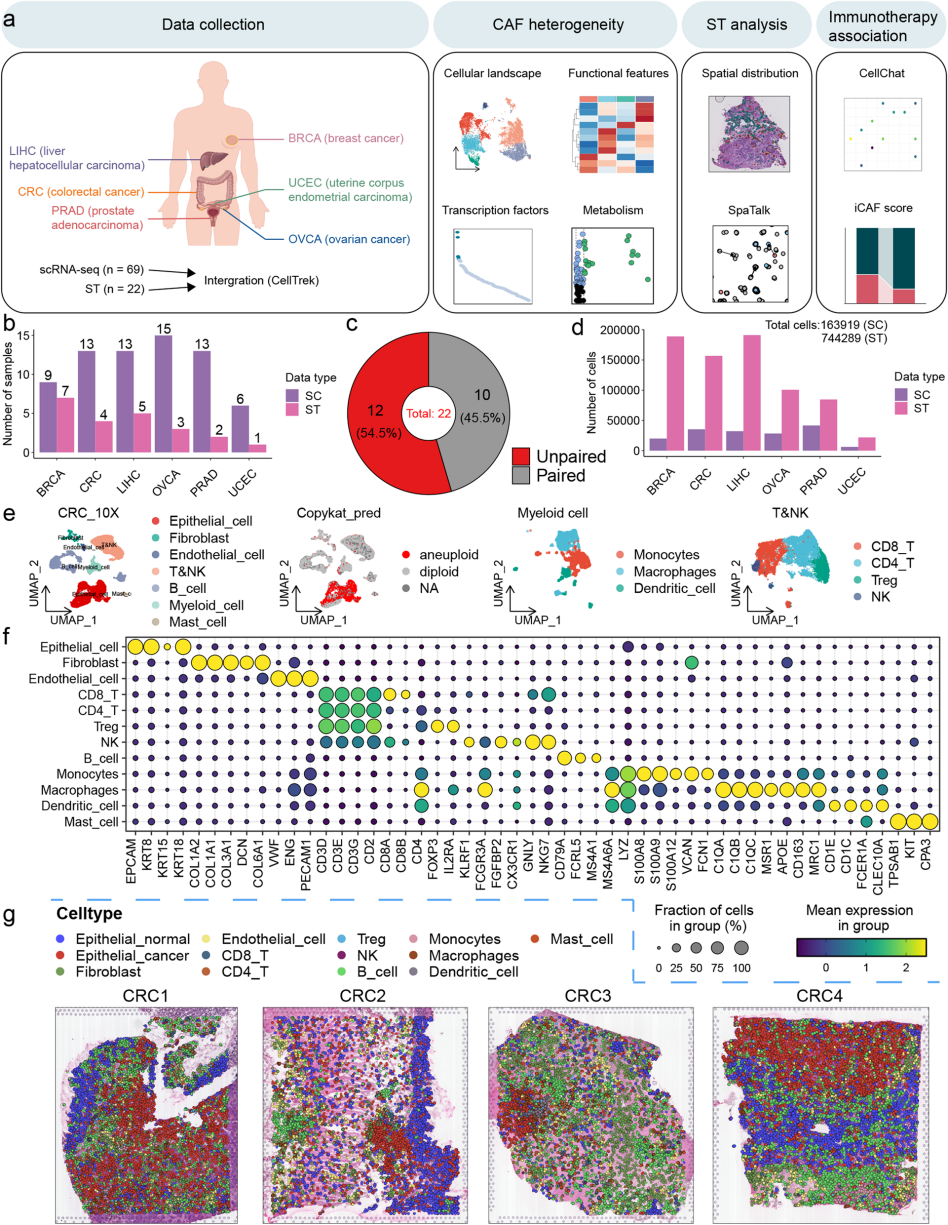
A pan-cancer spatial single-cell transcriptome atlas. **a** Schematic depicting the study design. The cancer types included in this pan-cancer study were displayed in the first image on the left, created by Figdraw. **b** The number of samples in the pan-cancer analysis of scRNA-seq and ST. **c** Pie chart showing the proportion of ST sections that have corresponding scRNA-seq data from the same patient compared to those without such corresponding scRNA-seq data. **d** The number of cells in the pan-cancer analysis of scRNA-seq and ST. **e** Uniform Manifold Approximation and Projection (UMAP) plots showing the major cell types in CRC. **f** Bubble heatmap showing the expression of marker genes for the major cell types in CRC. **g** Spatial cell charting of CRC using CellTrek

To establish a spatial single-cell landscape in pan-cancer, we acquired scRNA-seq data from 69 samples of 56 patients diagnosed with one of the six prevalent cancer types, along with ST data from 22 tissue slices of 22 patients (Fig. 1a and b; Table S1 and S2). Among them, the ST data of 10 tissue slices had corresponding scRNA-seq data from the same patient (Fig. 1c; Table S1 and S2). The data we collected included six types of cancer: BRCA, colorectal cancer (CRC), liver hepatocellular carcinoma (LIHC), ovarian cancer (OVCA), prostate adenocarcinoma (PRAD), and uterine corpus endometrial carcinoma (UCEC) (Fig. 1a; Table S1 and S2). After strict quality control and filtration, a total of 163,919 cells in the scRNA-seq data and 59,529 spots in ST data were retained for downstream analysis (Fig. 1d and S1a). In the scRNA-seq dataset, the median number of unique molecular identifiers (UMIs) per cell was 3955, and the median number of genes per cell was 1425 (Figure S1b and c). For ST analysis, the median number of UMIs per spot was 11,139 and the median number of genes per spot was 3,863 (Figure S1d and e). To minimize the batch effect between different scRNA-seq datasets, we independently analyzed each dataset. Taking CRC as an example, we used graph-based clustering and identified seven major clusters based on typical markers of different cell types (Table S3), including epithelial cells, fibroblasts, endothelial cells, T&NK, B cells, myeloid cells and mast cells (Fig. 1e and f). CopyKAT was used to estimate the single-cell copy number variation (CNV) landscape of tumors, in order to distinguish malignant epithelium from non-malignant epithelium (Fig. 1e). The myeloid cells were further divided into monocytes, macrophages and dendritic cells (Fig. 1e). The CD8 + T cells, CD4 + T cells, regulatory T cells (Treg cells) and natural killer cells (NK cells) were identified from the T&NK cluster (Fig. 1e). Similarly, cells from the other 5 types of cancer were clustered into roughly the same subgroups (Figure S2, S3, S4 and S5). Of note, we detected neutrophils in the scRNA-seq data from the inDrop platform, which were not detected in the scRNA-seq data from the 10 × Genomics platform (Figure S2). Neutrophils are very fragile and have low RNA content [13], which may be the main reason for the capture failure.

CellTrek is a computational toolkit that enables direct mapping of individual cells back to their spatial coordinates in tissue sections based on scRNA-seq and ST data [14]. Unlike ST deconvolution methods, this approach transferred ST coordinates to single cells, thereby achieving single-cell resolution [14]. We applied it to quality-controlled scRNA-seq and ST data in pan-cancer to reconstruct spatial single-cell atlases. Even without corresponding scRNA-seq data from the same patient, ST datasets were still largely covered by scRNA-seq datasets based on the co-embedding results (Figure S6). Due to some cells being repeatedly mapped, we ultimately obtained a pan-cancer spatial single-cell transcriptomic atlas containing 744,289 cells (Fig. 1d and g).

### CAF heterogeneity in pan-cancer

**Fig. 2:**
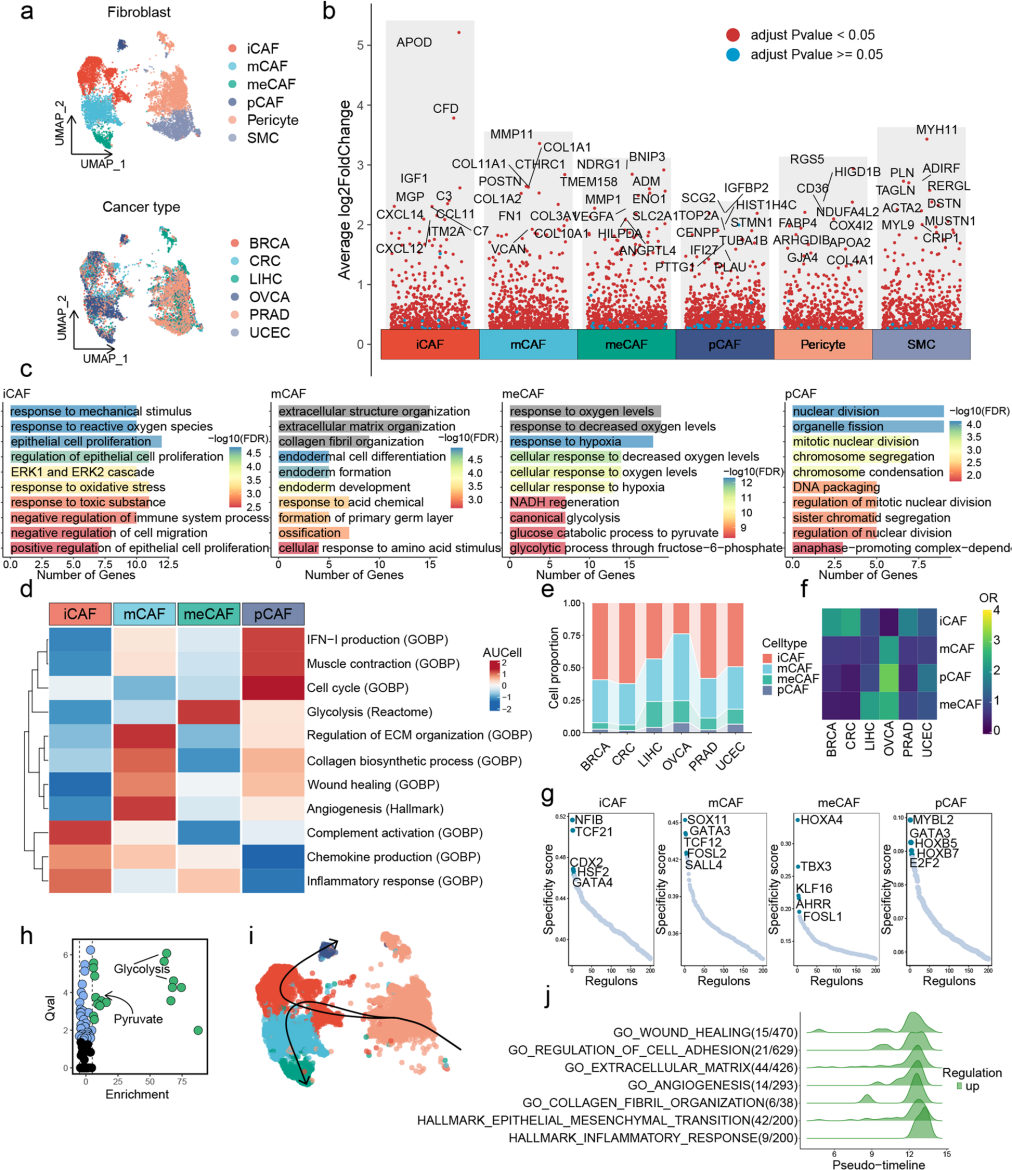
CAF heterogeneity in pan-cancer. **a** UMAP plots showing the integration of fibroblasts across six different cancer types by Harmony. **b** Differential expression analysis showing the upregulated genes for each fibroblast subtype. An adjusted *p* value < 0.05 is indicated in red, while an adjusted *p* value ≥ 0.05 is indicated in blue. **c** GO enrichment analysis of upregulated genes in each CAF subtype. **d** Heatmap showing pathway activities scored by AUCell in each CAF subtype. **e** Proportion of CAF subtypes across multiple cancer types. **f** Heatmap showing the ORs of CAF subtypes in each cancer type. **g** Scatter plot showing the RSSs in each CAF subtype. The top 5 regulons are highlighted. **h** SCAP analysis of metabolic pathways in meCAFs. **i** Slingshot trajectory analysis of CAFs. **j** GeneSwitches analysis of pathway activity changes in the transition pathway from pericytes to iCAFs

To compare the similarity of the main cell lineages of different cancer types, we constructed a phylogenetic tree (Figure S7a). Compared with the biased distribution of epithelial cells, fibroblasts from different cancer types clustered together (Figure S7a), indicating that fibroblasts had similar transcriptional features in different cancer types. Interestingly, NK cells and B cells originating from UCEC demonstrated unique features (Figure S7a), implying that the TMEs across diverse cancer types could have potentially exerted distinct effects on immune cell phenotypes. We subsequently investigated the heterogeneity of fibroblasts in scRNA-seq datasets of the 6 cancer types (Fig. 2a). The reclustering of the fibroblast cluster identified four CAF subtypes, as well as pericytes and smooth muscle cells (SMCs) (Fig. 2a). After applying Harmony for batch correction, all cells with local inverse Simpson's Index (LISI) greater than 1 indicate that no obvious batch effects were observed (Figure S8). CFD + fibroblasts showed high expression of chemokines (CCL11, CXCL12, and CXCL14) (Fig. 2b; Table S4), similar to the previously reported iCAFs in various types of tumors such as BC [6] and PTC [8]. GO enrichment analysis of its marker genes showed their association with the response to mechanical stimulation, reactive oxygen species, epithelial cell proliferation, immune system, and cell migration (Fig. 2c). POSTN + fibroblasts showed high expression levels of several ECM remodeling genes (MMP11, CTHRC1, COL1A1, COL1A2, COL3A1, COL10A1, and COL11A1) and enriched signatures of ECM (Fig. 2b and c; Table S4), which were consistent with the previously reported matrix CAFs (mCAFs) in cervical squamous cell carcinoma (CESC) [15]. Interestingly, a cluster of cells was related to the response to hypoxia and canonical glycolysis (Fig. 2c), resembling the reported metabolic CAFs (meCAFs) in pancreatic ductal adenocarcinoma (PDAC) [16]. Notably, we also found a cluster of cells that exhibited higher expression of a set of cell cycle-related genes (CENPF, NUSAP1, PTTG1, STMN1, TOP2A, and TUBA1B) (Fig. 2b; Table S4), which was consistent with proliferative CAFs (pCAFs) in a previous pan-cancer study of CAFs [10]. Immunofluorescence on tissue microarrays from BRCA patients further substantiated the existence of the four CAF subtypes (Figure S9). Next, we further investigated the heterogeneity of CAFs using the AUCell algorithm, based on the functional features of CAFs summarized by Lavie et al. [17] (Fig. 2d; Table S5). iCAFs exhibited the highest activity in immune-related functions, including complement activation, chemokine production, and inflammatory response (Fig. 2d). Additionally, the biological processes of angiogenesis, wound healing, regulation of ECM organization and collagen biosynthetic process were all enriched in mCAFs (Fig. 2d). As expected, meCAFs exhibited a high level of glycolytic activity (Fig. 2d). Interestingly, in addition to the cell cycle, pCAFs were also involved in IFN − I production and muscle contraction (Fig. 2d).

Although iCAFs and mCAFs were the major CAFs celltypes across 6 cancer types, different subtypes of CAFs still exhibited significant cancer preferences (Fig. 2e and f). iCAFs were enriched in BRCA and CRC, whereas meCAFs were enriched in LIHC and OVCA (Fig. 2f). The other two subtypes, especially pCAFs, were enriched in OVCA (Fig. 2f). To investigate the presence of these fibroblast subtypes in other common cancer types, we obtained and analyzed publicly available scRNA-seq data from non-small cell lung cancer (NSCLC) [18] and melanoma [19] (Figure S10a and b). Moreover, we conducted scRNA-seq on tumor and adjacent non-tumor tissues from a patient with HNSCC and integrated it with previously published scRNA-seq data of the same cancer type [7] (Figure S10c; Table S6). The findings indicated that both iCAFs and mCAFs were observed in all three types of cancer (Figure S10a-c). Next, we performed scRNA-seq on three samples derived from two patients with two rare types of tumors that have not been previously studied by scRNA-seq, including one tumor tissue from epithelial-myoepithelial carcinoma (EMC), one tumor tissue from mucoepidermoid carcinoma (MEC), and one adjacent non-tumor tissue from MEC (Figure S10d and e; Table S6). iCAFs and mCAFs were also both identified in these two types of tumors (Figure S10d and e). To investigate the key differences between fibroblasts derived from tumor tissue and adjacent non-tumor tissue, we conducted differential expression analysis. The results revealed a significant upregulation of multiple marker genes of mCAFs in fibroblasts derived from tumor tissues of HNSCC patients, as compared to fibroblasts derived from adjacent non-tumor tissues (Figure S10f). Functional enrichment analysis results indicated that the upregulated genes were related to the ECM, which was also observed in MEC (Figure S10g-i).

Via SCENIC analysis, we determined essential motifs within the four subtypes of CAFs. The regulatory protein CDX2 [20] associated with inflammation and the regulatory protein TCF12 [21] related to ECM remodeling were enriched in iCAFs and mCAFs, respectively (Fig. 2g; Table S7). Additionally, we observed that meCAFs were enriched for the KLF16 [22] (Fig. 2g; Table S7), which was a known regulator of metabolism. Lastly, Cell-cycle-related regulons (MYBL2 and E2F2) were highly enriched in pCAF (Fig. 2g; Table S7). The metabolic correlation of meCAFs prompted us to perform SCPA analysis to study their metabolic pathway activity. As expected, glycolysis and pyruvate were enriched in the top metabolic pathway of meCAFs (Fig. 2h; Table S8). Previous studies have reported that CAFs provide energy to cacner cells through glycolysis in hypoxic TME [23, 24]. This reverse Warburg effect may be caused by meCAFs. In order to further investigate metabolic reprogramming of CAFs, we conducted scMetabolism analysis and identified various metabolic rewiring mechanisms related to tumor growth in different CAF subgroups [24]. mCAFs exhibited higher activity in fatty acid biosynthesis, while the TCA cycle was enriched in pCAFs (Figure S11). Besides glycolysis, metabolism of alanine, aspartate, and glutamate was also enriched in meCAFs (Figure S11).

The complexity of CAF cellular characteristics can be attributed to their highly heterogeneous origins [17, 25]. In addition to transformation from tissue-resident fibroblasts, pericytes are also an important source for the formation of CAFs [17, 25]. With Slingshot analysis, a potential transition pathway from pericytes to iCAFs was suggested (Fig. 2i). Compared to other subtypes of CAFs, iCAFs exhibit the lowest level of transcriptional homogeneity (Figure S7b), which may be attributed to their complex origins. Previous studies have indicated that the transition from pericytes to fibroblasts is closely associated with cancer invasion and metastasis [26]. GeneSwitches analysis identified multiple biological processes that were activated along the pathway from pericytes to iCAFs, including wound healing, regulation of cell adhesion, ECM, angiogenesis, collagen fibril organization, epithelial-mesenchymal transition (EMT), and inflammatory response (Fig. 2j). While our data suggests that CAFs derived from pericytes are iCAFs, further investigation is necessary to explore its possibility and underlying mechanisms.

### Spatial distribution characteristics of CAFs

**Fig. 3:**
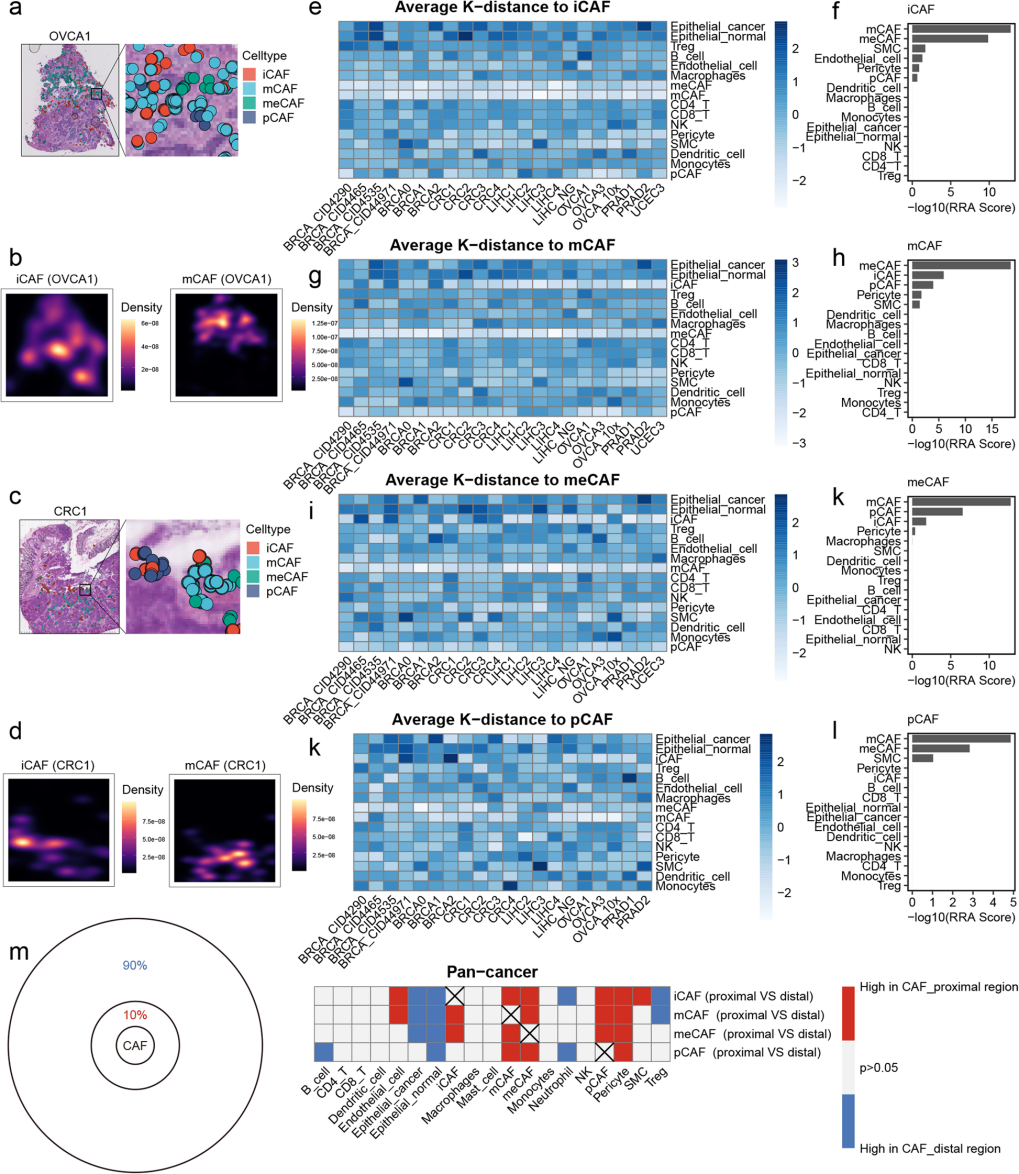
Spatial distribution characteristics of CAFs. **a** Spatial cell charting of CAFs in OVCA1 using CellTrek. **b** Density plots showing high-density regions of iCAFs and mCAFs in OVCA1. **c** Spatial cell charting of CAFs in CRC1 using CellTrek. **d** Density plots showing high-density regions of iCAFs and mCAFs in CRC1. **e** Heatmap showing the average k-distance from different cell types to iCAFs in each tissue tissue slice. The columns were scaled. **f** Integrated ranking of cell types based on proximity to iCAFs using RRA algorithm across 22 tissue slices. **g** Heatmap showing the average k-distance from different cell types to mCAFs in each tissue slice. The columns were scaled. **h** Integrated ranking of cell types based on proximity to mCAFs using RRA algorithm across 22 tissue slices. **i** Heatmap showing the average k-distance from different cell types to meCAFs in each tissue slice. The columns were scaled. **j** Integrated ranking of cell types based on proximity to meCAFs using RRA algorithm across 22 tissue slices. **k** Heatmap showing the average k-distance from different cell types to pCAFs in each tissue slice. The columns were scaled. **l** Integrated ranking of cell types based on proximity to pCAFs using RRA algorithm across 22 tissue slices. **m** Left: Circular plot showing the proportions of proximal and distal regions of CAFs. Right: heatmap showing the enrichment of various cell types in the proximal and distal regions for each CAF subpopulation. The paired t-test was used to compare the differences in cell proportions between the proximal and distal regions for each CAF subpopulation. Red color represents enrichment of a cell type in the proximal region of CAFs, while blue color represents enrichment of a cell type in the distal region of CAFs. Only *p* values < 0.05 are shown

To determine the spatial distribution characteristics of CAFs, we added their cell subpopulation annotation information into the CellTrek object. As the cell ratio of meCAFs and pCAFs is very low, we first focused our analysis on iCAFs and mCAFs. Taking one tissue section each from OVCA (OVCA1) and CRC (CRC1) as examples, we observed a spatially exclusive phenomenon between the high-density areas of iCAFs and mCAFs (Fig. 3a-d), suggesting that the activation state of CAFs is related to their location within the TME. To dissect the spatial expression dynamics from high-density areas of iCAFs to high-density areas of mCAFs, we conducted spatial trajectory analysis in OVCA1 and CRC1. The results demonstrated a gradual change in the proportions of cells along the trajectory, accompanied by a gradual increase in features such as collagen biosynthetic process, regulation of ECM organization, wound healing, and angiogenesis (Figure S12a-f). In addition, our slingshot analysis revealed a potential transition path from iCAFs to mCAFs (Fig. 2i), which is consistent with previously reported lineage plasticity among CAF subpopulations [17]. Overall, these results suggest that the state of CAFs could potentially be influenced by the specific TME.

Robust rank aggregation (RRA) is an algorithm that integrates ranks to obtain a comprehensive ranking list [27]. We computed the spatial k-distance between all cells and the subpopulations of fibroblasts in each tissue section, sorted them from closest to farthest, and integrated them using the RRA algorithm to obtain a comprehensive ranking of all cells. Upon analysis, the four subtypes of CAFs exhibited the minimum spatial k-distance between them, whereas there were no notable differences in the ordering of immune cell subtypes (Fig. 3e-l). To further investigate the microenvironmental characteristics surrounding different CAF subtypes, cells within the top 10% of spatial k-distance from the fibroblasts were defined as "CAF-proximal cells", with all others classified as "CAF-distal cells" (Fig. 3m). Then, through paired t-tests, we compared the proportion of cells between CAF-proximal and CAF-distal cells. As anticipated, there was an enrichment of other types of CAF subtypes surrounding each type of CAF subtype (Fig. 3m). Additionally, a higher density of pericytes was observed in the vicinity of CAFs (Fig. 3m), which serve as an important source of CAFs. Endothelial cells exhibited an increased abundance in proximity to iCAFs and mCAFs (Fig. 3m). This observation may be explained by the angiogenic effect of mCAFs and the potential transformational relationship between iCAFs and mCAFs. Notably, the proportion of epithelial cells decreased around all four CAF subtypes (Fig. 3m), implying that these subtypes are located farther away from the epithelial area. Moreover, a decrease in the proportion of certain immune cell subtypes was observed in the vicinity of CAFs, including a reduction in neutrophils and Tregs proportions around iCAFs, a decrease in Tregs proportions near mCAFs, and a lower proportion of B cells near pCAFs (Fig. 3m).

### Effect of CAFs on TME through paracrine signaling

**Fig. 4:**
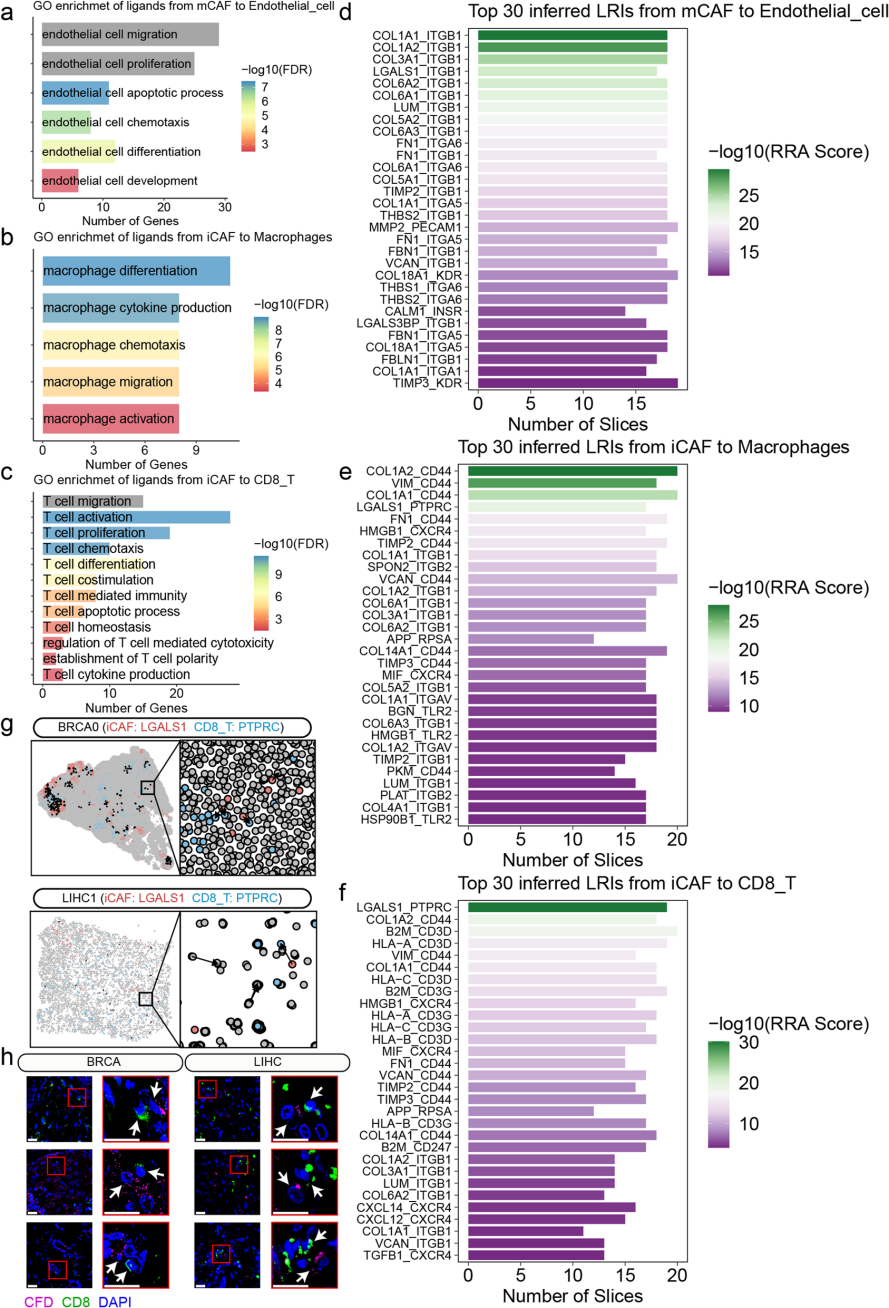
Effect of CAFs on TME through paracrine signaling. **a** GO enrichmet of ligands from mCAFs to endothelial cells. **b** GO enrichmet of ligands from iCAFs to macrophages. **c** GO enrichmet of ligands from iCAFs to CD8 + T cells. **d** Integrated ranking of LRIs based on number of LRIs from mCAFs to endothelial cells using RRA algorithm across 22 tissue slices. **e** Integrated ranking of LRIs based on number of LRIs from iCAFs to macrophages using RRA algorithm across 22 tissue slices. **f** Integrated ranking of LRIs based on number of LRIs from iCAFs to CD8 + T cells using RRA algorithm across 22 tissue slices. **g** Spatial distribution of the LGALS1-PTPRC interaction on two spatial transcriptomics tissue slices (BRCA0 and LIHC1). **h** Representative immunofluorescence images of CFD (red) and CD8 (green) in tissues from three patients with BRCA and three patients with LIHC. Scale bar represents 20 μm

Given the high angiogenic activity of mCAFs, we employed Spatalk to explore the interplay between mCAFs and endothelial cells within the spatial context. Angiogenesis is a complex molecular process involving endothelial cell activation, proliferation, and migration to form new blood vessels and vasculature [28, 29]. Intriguingly, the ligands identified in mCAFs were found to play significant roles in various endothelial cell functions, including migration, proliferation, apoptosis, chemotaxis, differentiation, and development (Fig. 4a and S13). Our analysis further revealed a series of ligand-receptor interactions (LRIs) associated with angiogenesis, such as VEGFA-(FLT1 + KDR + NRP1 + ITGB1 + ITGA9), VEGFB-(FLT1 + NRP1), VEGFC-(FLT1 + KDR + ITGB1), PGF-(NRP1 + FLT1), and THBS1-(ITGA6 + ITGB1 + LRP5) [30, 31] (Fig. 4d and S13; Table S9). Collectively, these findings suggest that mCAFs exert their pro-angiogenic effects by modulating endothelial cell function through paracrine signaling.

In addition to their impact on angiogenesis within TME, CAFs also regulate immune cell responses to promote tumor growth and immune escape [17]. Ligands derived from iCAFs to macrophages were found to significantly enrich various macrophage functions, including differentiation, cytokine production, chemotaxis, migration, and activation (Fig. 4b and S14). Considering the close association between M2 macrophage polarization and tumor progression, our investigation focused on the influence of iCAFs on M2 macrophage polarization. We collected relevant ligands based on previous findings [32] (Table S10) and identified a series of LRIs involved in this process, such as TGFB1- (CXCR4 + ITGAV + TGFBR2 + TGFBR1 + ITGB5 + SDC2 + SMAD3 + ITGB8), TGFB2-(TGFBR2 + TGFBR1 + ACVR1), TGFB3-(ITGB1 + TGFBR2 + ITGB5 + TGFBR1), CSF1-(CSF1R + SIRPA), IL34-CSF1R, and IL10-(IL10RA + IL10RB) [32, 33] (Fig. 4e and S14; Table S9).

CD8 + T cells play a pivotal role in anti-tumor immunity, yet the mechanisms underlying the interaction between iCAFs and CD8 + T cells remain elusive. Ligands from iCAFs that bind to CD8 + T cells were enriched in various T cell-related functions, including migration, activation, proliferation, chemotaxis, differentiation, costimulation, apoptotic process, homeostasis, cytokine production, establishment of T cell polarity, T cell mediated immunity and cytotoxicity (Fig. 4c and S15). Notably, iCAFs may induce CD8 + T cells apoptosis and impair their anti-tumor functions by interacting with CD8 + T cells PTPRC receptors via Galectin-1 (LGALS1) [34, 35] (Fig. 4f and S15; Table S9). Additionally, iCAFs can suppress the activation and proliferation of CD8 + T cells through macrophage migration inhibitory factor (MIF)-CXCR4 interaction [36] (Fig. 4f and S 15; Table S9). Furthermore, iCAFs may secrete TGFB1 to inhibit the activation and proliferation of CD8 + T cells [37] (Fig. 4f and S15; Table S9). Apart from their interactions with CD8 + T cells and macrophages, iCAFs exhibited complex interplays with other immune cell populations, including B cells, dendritic cells, mast cells, neutrophils, NK cells, and Tregs (Figure S16a-l; Table S9). These comprehensive findings highlight the critical role of iCAFs in shaping the immunosuppressive microenvironment.

Given the crucial role of LGALS1 in tumor immune evasion [38], our subsequent analysis focused on the LGALS1-PTPRC interaction, which was observed in ST tissue slices of various tumors (Fig. 4g). Immunofluorescence experiments on tissue microarrays of BRCA and LIHC also unveiled a multitude of instances wherein CFD-positive cells and CD8-positive cells exhibited spatial proximity (Fig. 4h). By analyzing datasets derived from The Cancer Genome Atlas (TCGA) database, we observed a significant positive correlation between LGALS1 and PDCD1 in five tumor types, with the exception of UCEC (Figure S17a). Notably, we identified nuclear factor of activated T cells 1 and 2 (NFATC1 and NFATC2) in the intracellular signaling network triggered by LGALS1- PTPRC interaction (Figure S18a), which are considered as key transcription factors (TFs) leading to CD8 + T cells exhaustion [39, 40]. To investigate the association between NFATC1/2 and CD8 + T cells exhaustion, we analyzed CD8 + T cells from pan-cancer scRNA-seq data. CD8 + T cells were re-clustering into thirteen subpopulations, and Slingshot analysis identified six distinct lineages (Figure S18b and c). The C6 cluster, marked by high expression of naive markers including CCR7 and TCF7, was deemed as the trajectory starting point (Figure S18d). The C8 cluster was characterized by upregulation of T cell exhaustion markers (HAVCR2, TIGIT, LAG3, PDCD1, CXCL13, and LAYN) and identified as exhausted CD8 + T (Tex) cells (Figure S18d). Confirming our expectations, GeneSwitches analysis revealed the activation of NFATC2 following immune checkpoint gene induction in the T cell exhaustion trajectory (lineage 1) (Figure S18e). Moreover, our analysis of the TCGA datasets also revealed a significant positive correlation between NFATC2 and PDCD1 across six distinct tumor types (Figure S17b). Collectively, these findings expand our understanding of the role of iCAFs in mediating CD8 + T cells exhaustion.

### Anti-PD1 treatment influences the communication between iCAFs and TME

**Fig. 5:**
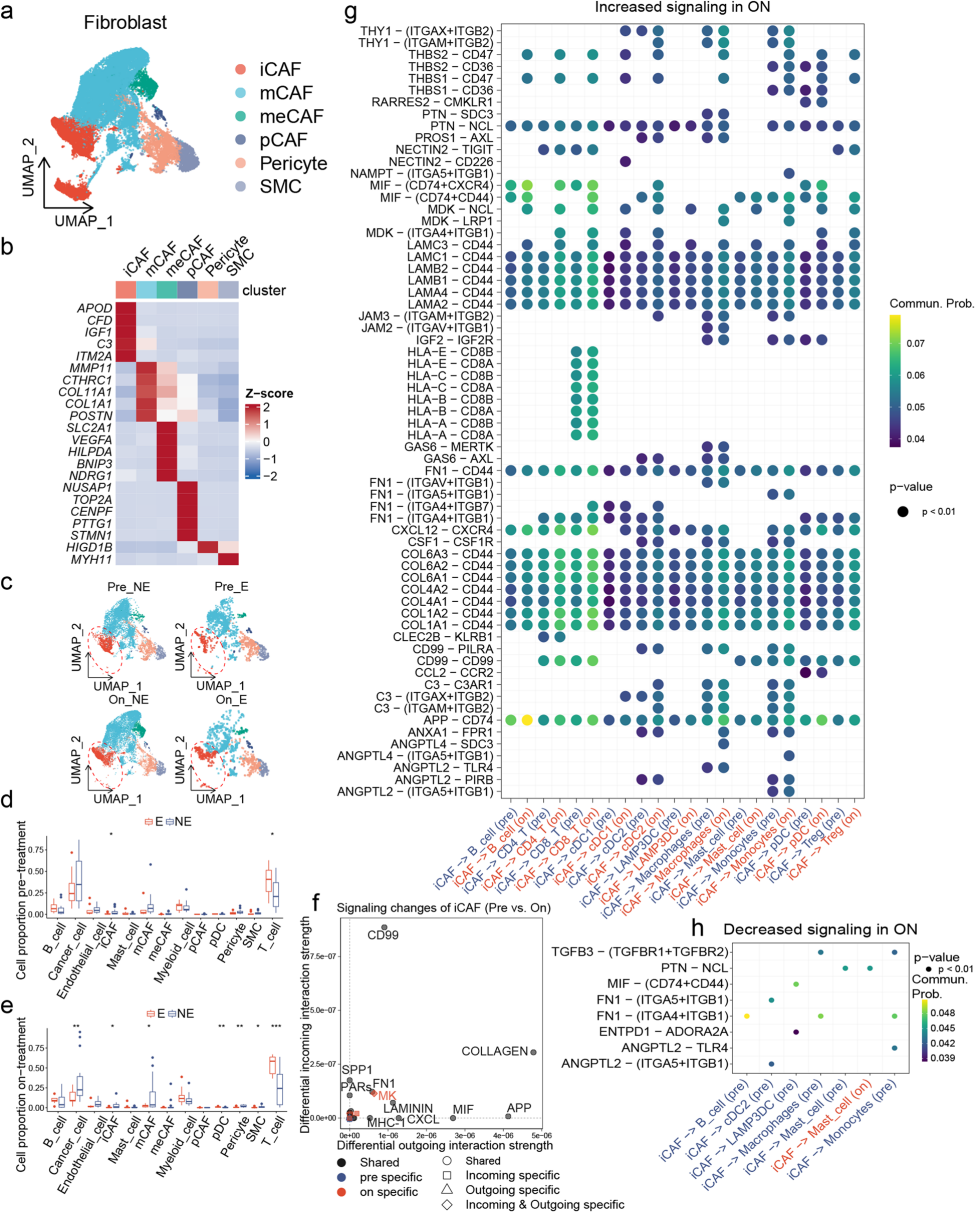
Anti-PD1 treatment influences the communication between iCAFs and TME. **a** UMAP plot showing the fibroblasts subpopulations in BRCA immunotherapy cohort. **b** Heatmap showing the expression of marker genes in fibroblast subpopulations. **c** UMAP plots showing the temporal alterations of fibroblasts subpopulations. **d** Boxplot showing the differences in cell proportions between patients with and without clonal expansion before anti-PD1 treatment. Statistical analysis was performed using unpaired t-tests; *P < 0.05, **P < 0.01, ***P < 0.001. **e** Boxplot showing the differences in cell proportions between patients with and without clonal expansion on anti-PD1 treatment. Statistical analysis was performed using unpaired t-tests; *P < 0.05, **P < 0.01, ***P < 0.001. **f** Differential cell–cell interaction signaling pathway alterations in iCAFs during anti-PD-1 treatment compared to pre-treatment. **g** Upregulated LRIs in iCAFs during anti-PD-1 treatment compared to pre-treatment. **h** Downregulated LRIs in iCAFs during anti-PD-1 treatment compared to pre-treatment

To investigate the effect of anti-PD1 therapy on iCAFs, we analyzed publicly available scRNA-seq data from 31 paired pre- and on-treatment samples of BRCA patients receiving pembrolizumab [41]. Interestingly, we obtained CAF subtyping results consistent with those in pan-cancer after further subclustering of fibroblasts (Fig. 5a and b). We then stratified the samples based on T-cell clonal expansion and treatment time point and compared the changes in cell proportions. Due to the absence of clonal expansion information for two patients, they were excluded from this analysis. During treatment, patients with clonal expansion had lower proportions of cancer cells compared to those without, potentially due to an increase number of T cells with cytotoxic activity (Fig. 5e). Moreover, the proportion of iCAFs was consistently lower in patients with clonal expansion compared to those without, both pre- and on-treatment (Fig. 5c-e). Notably, for both clonal expansion and non-clonal expansion patients, the proportion of iCAFs did not change during treatment compared to pre-treatment (Figure S19a and b).

While the proportion of iCAFs remained unchanged after anti-PD-1 treatment, it is possible that their transcriptional profiles underwent changes. To explore this possibility, we conducted differential expression analysis. Interestingly, we found that Chitinase-3-Like-1 (CHI3L1) was significantly upregulated (Figure S19c), which is a known regulator promoting M2 macrophage polarization [42]. Consistently, AUCell analysis revealed an enhanced ability of iCAFs to promote M2 macrophage polarization during the treatment compared to before (Figure S19e). It is noteworthy that the differentially expressed genes (DEGs) of iCAFs between pre-treatment and on-treatment were enriched in the TNFα signaling via NF-kB, epithelial cell proliferation, and EMT both before and during the treatment (Figure S19d). Similarly, the AUCell scores of epithelial cell proliferation and EMT in iCAFs were significantly increased during the treatment (Figure S19e). Additionally, the AUCell analysis results showed that anti-PD1 treatment also enhanced the complement activation feature of iCAFs (Figure S19e). Overall, these findings suggest that anti-PD1 treatment influences the communication between iCAFs and other cells, including promoting epithelial cell proliferation, EMT, and M2 macrophage polarization.

We next sought to determine the differences in communication between iCAFs and immune cells before and during anti-PD-1 therapy. We further categorized myeloid cells into monocytes, macrophages, LAMP3 + dendritic cells (LAMP3 + DCs), classical type 1 dendritic cells (cDC1s), and classical type 2 dendritic cells (cDC2s), and T cells into CD4 + T cells, CD8 + T cells, and Tregs, and conducted CellChat analysis between iCAFs and immune cells (Figure S19f-i). Notably, we found anti-PD1 treatment enhanced iCAFs' ability to promote the formation of an immunosuppressive microenvironment. Compared to pre-treatment, iCAFs secreted MIF and laminins during treatment to suppress the activation, proliferation, and migration of CD8 + T cells [36, 43–45] (Fig. 5f and g). Although iCAFs downregulated TGFB3 during treatment compared to pre-treatment, they may still promote monocyte survival and differentiation into tumor-associated macrophages by overexpressing macrophage colony-stimulating factor-1 (CSF-1) [46–48] (Fig. 5g and h). Through the CXCL12/CXCR4 axis, iCAFs may reduce CD8 + T cell infiltration, promote CD8 + T cell dysfunction, and increase the number of Tregs [49] (Fig. 5g).

### iCAF score correlate with immunotherapy response

**Fig. 6:**
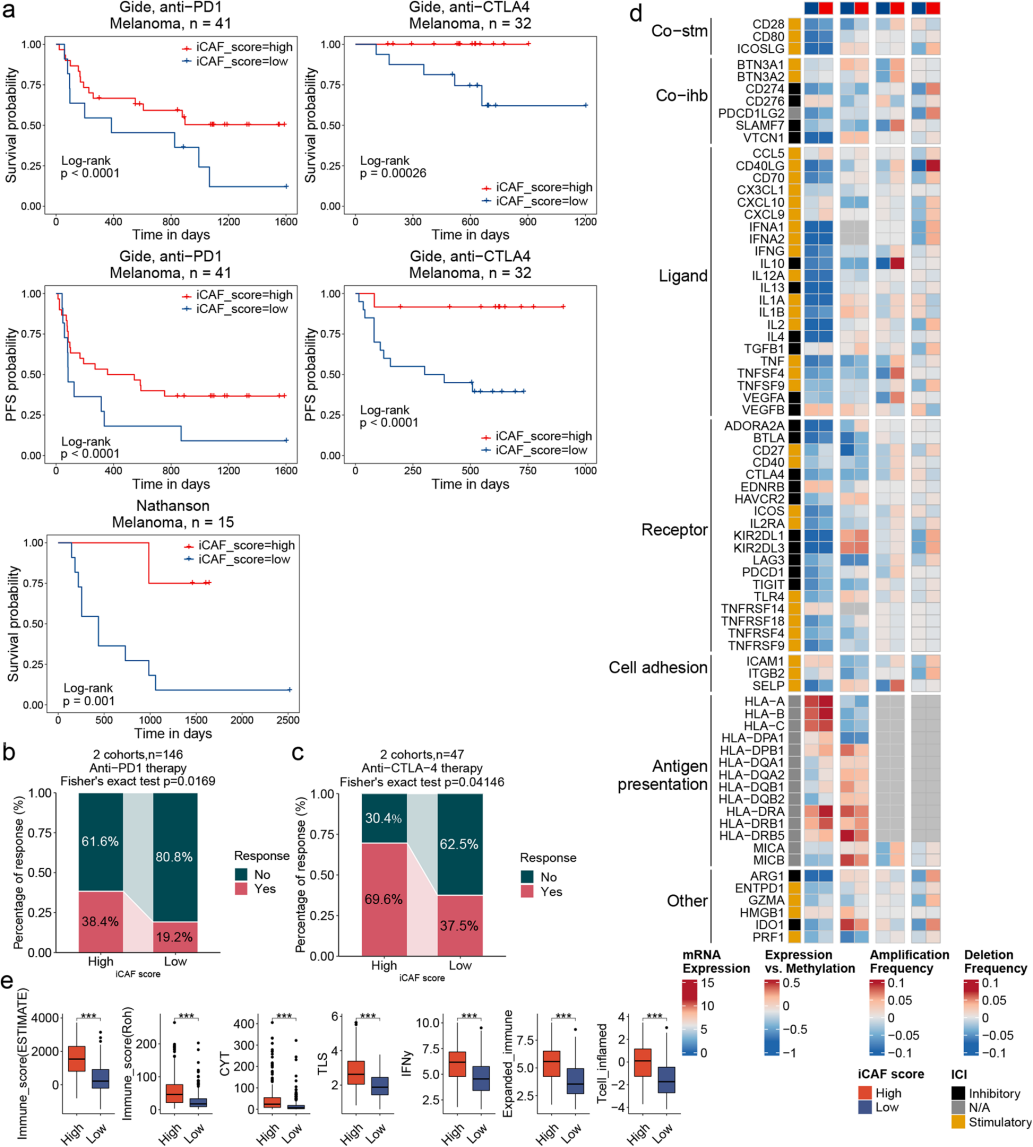
iCAF score correlate with immunotherapy response. **a** Kaplan–Meier plots showing the prognostic value of iCAF score in the melanoma immunotherapy cohorts. *P*-values were calculated by log-rank test. **b** Percentage of anti − PD1 therapy response among melanoma patients with high and low iCAF scores. Statistical analysis was performed using Fisher's exact test. **c** Percentage of anti − CTLA − 4 therapy response among melanoma patients with high and low iCAF scores. Statistical analysis was performed using Fisher's exact test. **d** Heatmap showing immune modulators in melanoma patients with high and low iCAF scores. From left to right: mRNA expression (median-normalized expression levels of immune modulators); expression versus methylation (Spearman correlation between expression of immune modulators and DNA methylation beta-values); amplification frequency (difference in the proportion of immune modulators amplifications between patients with high or low iCAF scores and the proportion of immune modulators amplifications in all patients.); and the deletion frequency (as amplifications). **e** Boxplot showing the comparison of immune related scores in melanoma patients with high and low iCAF scores. Statistical analysis was performed using Wilcoxon rank-sum tests; *P < 0.05, **P < 0.01, ***P < 0.001

Given the complex interplay between iCAFs and immune cells in TME, we hypothesized that the gene expression features of iCAFs are associated with immune checkpoint blockade (ICB) response. Using ssGSEA algorithm, we constructed an iCAF score using the top ten marker genes of iCAFs (Table S3) and applied it to different melanoma immunotherapy cohorts. In all cohorts, patients with high iCAF scores displayed prolonged overall survival (OS) (Log-rank test *P* < 0.0001 for the Gide anti-PD-1 cohort; *P* = 0.00026 for the Gide anti-CTLA-4 cohort and *P* = 0.001 for the Nathanson cohort; Fig. 6a) and progression-free survival (PFS) (Log-rank test *P* < 0.0001 for the Gide anti-PD-1 cohort and *P* < 0.0001 for the Gide anti-CTLA-4 cohort; Fig. 6a). Next, we divided the melanoma patients into high and low iCAF score groups based on the median and compared the percentage of ICB responders between the two groups. The results showed that patients with high iCAF scores had higher percentages of responders to anti-PD-1 treatment (Fisher's exact test *P* = 0.0169; Fig. 6b) and anti-CTLA-4 treatment (Fisher's exact test *P* = 0.04146; Fig. 6c). Moreover, consistent with these findings, both anti-PD-1 and anti-CTLA-4 responders had higher iCAF scores than non-responders (Figure S20a). These findings indicate that the iCAF score is a valuable tool in predicting patient survival and response to ICB therapy.

Tumor mutational burden (TMB) serves as a widely recognized biomarker for immunotherapy and is generally associated with patients' response to ICB [50, 51]. Therefore, we conducted a comprehensive analysis using data from TCGA database focusing on melanoma patients. Surprisingly, we found that melanoma patients with a high iCAF score exhibited significantly lower TMB compared to those with a low iCAF score (Figure S20b and c), suggesting the presence of additional mechanisms driving anti-tumor immune responses in high iCAF score melanoma patients. A previous study reported that TMB is not associated with the response to immunotherapy in melanoma patients [52]. Notably, apart from HYDIN and ADGRV1 mutations being more frequent in low iCAF score melanoma patients, there were no significant differences in the prevalence of other common mutations between high and low iCAF score groups (Figure S20b). Interestingly, we observed that the burden of CNVs at the arm level showed no significant difference between high and low iCAF score patients (Figure S20d). However, when examining CNVs at the focal level, we found that high iCAF score patients exhibited a lower burden of gain and loss of CNVs (Figure S20d). This pattern closely resembles the immune-rich tumor phenotype previously reported in LIHC [53] and CRC [54]. Based on these intriguing findings, we focused our analysis on the immune landscape of melanoma patients and found several key features associated with high iCAF score melanoma patients. Specifically, we observed higher expression of immune checkpoint genes (PDCD1, CTLA4, and LAG3) and a higher frequency of CNV amplifications in these patients (Fig. 6d and S20e). Notably, the majority of immune modulators showed elevated expression in high iCAF score patients (Fig. 6d and S20e), implying the presence of more complex interactions within the TME of these patients. The immune scores calculated by the ESTIMATE algorithm and previously reported immune response scores, including immune score (Roh) [55], cytolytic activity (CYT) [56], tertiary lymphoid structures signature (TLS) [57], IFNy [58], expanded immune [58], and T cell inflamed [58] (Table S11), were also found to be higher in patients with high iCAF scores (Fig. 6e). Furthermore, immune cells and multiple inflammatory pathways (JAK − STAT, NFkB, and *TNFa)* were enriched in high iCAF score patients (Figure S20f and g). These data suggest that the benefit of high iCAF score patients in tumor immunotherapy may rely on increased immune cell infiltration and the intricate interplay of immune modulators.

## Discussion

We have collected scRNA-seq data and ST data from patients with six prevalent cancer types to conduct a comprehensive study on the biological characteristics of CAFs in TME. While the proportions of distinct CAF subtypes varied among different cancer types, both iCAFs and mCAFs consistently emerged as the primary subtypes across all common cancer types. Interestingly, our observations extended to two rare cancer types, EMC and MEC, where the presence of iCAFs and mCAFs was also found.

It's worth noting that we also made an intriguing discovery regarding pCAFs, which exhibited heightened activity in IFN-I production. The role of IFN-I (Type I interferon) in cancer presents a dual-edged sword effect [59–63]. Acute exposure to high concentrations of IFN-I can lead to the growth arrest and apoptosis of cancer cells, whereas prolonged exposure to low concentrations of IFN-I may promote the survival of cancer cells [60]. Additionally, IFN-I plays a critical role in facilitating cDC1 cross-priming and CD8 + T cell reactivation [61, 63]. Therefore, a promising treatment strategy could involve combination immunotherapy targeting pCAF.

As widely recognized, metabolic reprogramming serves as a crucial hallmark of cancer cells, facilitating the establishment of a tumor-promoting microenvironment [64]. Recent studies have shed light on the impact of CAFs on cancer cell metabolism through their intrinsic metabolic reprogramming [24]. However, these studies often overlook the heterogeneity of CAFs, merely revealing the average metabolic characteristics across all subtypes. Employing a comprehensive pan-cancer single-cell analysis, we found diverse metabolic reprogramming mechanisms within distinct CAF subpopulations. Specifically, mCAFs exhibited enrichment in fatty acid biosynthesis, pCAFs displayed enrichment in the TCA cycle, while meCAFs demonstrated metabolic enrichment in glycolysis, alanine, aspartate, and glutamate metabolism. Consequently, the development of therapeutic strategies targeting the metabolic reprogramming of CAFs should consider the distinct characteristics exhibited by various subtypes of CAFs.

Pericytes are vital mural cells that can undergo pericyte–fibroblast transition (PFT) under the influence of changes in matrix stiffness and tumor-secreted factors [65, 66]. This phenotypic transition plays a crucial role in promoting tumor growth and metastasis [26]. In this study, we have uncovered a pericyte-iCAF transition pathway, suggesting that the initial fibroblasts derived from pericytes may be iCAFs. Along the transition pathway from pericytes to iCAFs, expression of genes associated with inflammatory response and ECM was significantly upregulated, indicating a potential involvement of PFT in facilitating the formation of an immunosuppressive microenvironment and ECM remodeling. Further exploration is warranted to unravel the functional roles and underlying mechanisms of PFT in this context.

In the spatial analysis, the four subpopulations of fibroblasts demonstrated a relatively closer spatial proximity compared to other cell types. We found an enrichment of endothelial cells in close proximity to mCAFs, and their intercellular communication was found to promote angiogenesis within TME. while CD8 + T cells were not found to be enriched in close proximity to iCAFs, we observed numerous instances of iCAFs-CD8 + T cells co-localization in situ across various tumor types, which was further confirmed by immunofluorescence. The complex interplay between iCAFs and immune cells, particularly macrophages and CD8 + T cells, facilitates the establishment of an immunosuppressive microenvironment. Notably, analyzing scRNA-seq data from BRCA patients receiving anti-PD-1 immunotherapy, we have identified that anti-PD-1 immunotherapy enhances the capacity of iCAFs to promote the establishment of an immunosuppressive microenvironment. Additionally, there was a significant correlation observed between the iCAF score constructed based on iCAF marker genes and the immune therapy response in melanoma patients. Therefore, the combination of targeted interventions against iCAFs with anti-PD-1 treatment holds promising potential as a valuable therapeutic approach.

## Conclusion

In conclusion, our comprehensive analysis of pan-cancer spatial and single-cell data has unraveled the heterogeneity of CAFs, shedding light on their spatial distribution patterns and intricate cell communication with TME. To facilitate further exploration of CAF heterogeneity, we have developed an interactive website (https://chenxisd.shinyapps.io/pancaf/) using the ShinyCell R package [67]. Our pan-cancer study not only enhances our understanding of CAF biological characteristics but also provides important insights for the development of targeted therapeutic approaches aimed at CAFs.

## Methods

### Sample acquisition and processing

The study was approved by the Ethics Committee of Qilu Hospital of Shandong University (KYLL-2017–256) and conducted in accordance with the Declaration of Helsinki. All subjects gave written informed consent before participating in the study. One sample of MEC, one sample of EMC, one sample of HNSCC and two non-malignant samples (control group, one adjacent normal tissue from MEC and one adjacent normal tissue from HNSCC), were obtained from the Qilu Hospital of Shandong University, Jinan, China. The clinical information for these samples is provided in Table S6. All samples were processed immediately after being obtained from oral cancer surgery according the standard procedures. According to the manufacturer's instructions, the Human Tumor Dissociation Kit (Miltenyi Biotec; Order no: 130–095-929) was used to obtain single cells from the tissues.

### Single-cell RNA sequencing

According to the manufacturer’s protocol, Chromium Single cell 3′ Reagent v3 kits were used to prepare barcoded scRNA-seq libraries. The cell concentration was adjusted to 700–1200 cells/μL. The gel beads, carrying barcode information, were combined with a mixture of cells and enzymes, and subsequently enveloped by oil droplets, forming gel beads in emulsions (GEMs). The gel beads within GEMs underwent dissolution, releasing mRNA upon cell lysis. Reverse transcription was then performed to generate barcoded cDNA for sequencing. After disrupting the liquid oil layer, cDNA amplification was carried out, followed by purification and quality inspection. Subsequently, the cDNA was digested into fragments of approximately 200–300 bp, and then subjected to the traditional second-generation sequencing library construction process, which included the addition of sequencing adapter P5 and sequencing primer R1, followed by PCR amplification to obtain the DNA library. Finally, the constructed library was subjected to high-throughput sequencing using the Illumina NovaSeq 6000 platform in a paired-end sequencing mode.

### scRNA-seq data and ST data processing

The newly generated raw scRNA-seq data were processed by CellRanger (v 3.1.0) to generate a UMI count matrix. The human genome (hg38) was used as a reference. Raw gene expression matrices were constructed into a Seurat object and imported into R software by Seurat R package [68]. Low-quality cells (> 40,000 UMI/cell, < 500 genes/cell, > 5,000 genes/cell and > 20% mitochondrial genes) were excluded. Doublets were identified and removed by DoubletFinder R package [69]. The harmony R package [70] was utilized for batch effect correction. We utilized the local inverse Simpson's Index (LISI) to evaluate batch effects [70]. We performed principal component analysis (PCA) to reduce the dimensionality of scRNA-seq data. Top 30 principal components (PCs) were selected for UMAP. The FindClusters function was used to identify cell clusters.

We applied the same processing pipeline to publicly available scRNA-seq datasets from the 10 × Genomics platform. For the public scRNA-seq datasets sourced from the inDrop platform, we performed quality control by filtering out cells with UMI counts greater than 40,000, cells with gene counts less than 200, cells with gene counts exceeding 5000, and cells with mitochondrial gene count surpassing 30%.

We imported the publicly available ST dataset into Seurat using the Load10X_Spatial function. Subsequently, we filtered out low-quality spots with gene counts below 500 and mitochondrial gene count exceeding 30%.

### Recognition of malignant and non-malignant epithelial cells

The copykat R package [71] was used to identify malignant and non-malignant epithelial cells with default parameters. The cells from TME were used as a normal reference.

### CellTrek analysis

To acquire the spatial coordinates of the cells, we conducted a combined analysis of the scRNA-seq data and ST data using the CellTrek R package [14] with its default parameters.

We utilized the run_kdist function from the CellTrek package to calculate the spatial k-distance between different cell types. The analysis followed the parameters: ref_type = "all", keep_nn = F, k = 10.

### Comparison dendrograms

To conduct a phylogenetic analysis of the different cell subpopulations within the pan-cancer scRNA-seq dataset, we utilized the BuildClusterTree function from the Seurat R package. To visualize the results, the ggtree R package [72] was applied.

### Differential expression analysis and functional enrichment analysis

To identify DEGs in the scRNA-seq data, we utilized the "FindAllMarkers" or "FindMarkers" functions in Seurat. The thresholds were set as |log2FC|> 1 and adj.p.val < 0.05. Subsequently, we conducted functional enrichment analysis of the DEGs using the WebGestaltR R package [73]. For this analysis, we selected the "genome protein-coding" as the reference set.

### Cancer preferences analysis

To assess the cancer preferences of CAF subtypes, odds ratios (OR) were calculated using the computational method described by Zhang et al. [74]. This involved constructing a 2 by 2 contingency table for each combination of CAF subtypes i and cancer types j. The table included the number of cells from CAF subtypes i in cancer types j, the number of cells from CAF subtypes i in other cancer types, the number of cells from non-i CAF subtypes in cancer types j, and the number of cells from non-i CAF subtypes in other cancer types. Fisher's exact test was then performed on the contingency table.

### SCENIC analysis

To calculate the regulon activity scores (RAS) of CAFs, we used the pySCENIC Python package [75] for SCENIC analysis. First, GRNBoost2 was used to infer the co-expression modules between TFs and candidate target genes. Then, RcisTarget was used to analyze the genes in each co-expression module to identify the enriched motifs (a TF and its potential direct target genes were defined as a regulon). Finally, AUCell was used to evaluate the activity of each regulon in each cell.

We measured the cell-type specificity of a regulon by calculating the regulon specificity score (RSS) using the computational method described by Suo et al. [76]. First, we defined a probability distribution of RAS ${P}^{R}=\left({P}_{1}^{R}, \dots , {P}_{n}^{R}\right)$ and normalized it so that $\sum_{i=1}^{n}{P}_{i}^{R}=1$. Second, we defined a probability distribution of cell types ${P}^{C}=\left({P}_{1}^{C}, \dots , {P}_{n}^{C}\right)$ to indicate whether a cell belongs to a specific cell-type (${P}_{i}^{C}=1$) or not (${P}_{i}^{C}=0$) and normalized it so that $\sum_{i=1}^{n}{P}_{i}^{C}=1$. Then, we calculated the Jensen-Shannon Divergence (JSD) $JSD\left({P}^{R},{P}^{C}\right)=H\left(\frac{{P}^{R}+{P}^{C}}{2}\right)-\frac{H\left({P}^{R}\right)+H({P}^{C})}{2}$ to measure the difference between the two probability distributions. Finally, RSS was calculated as: $RSS\left(R,C\right)=1-\sqrt{JSD\left({P}^{R},{P}^{C}\right)}$.

### Gene set scoring

To score gene sets in the scRNA-seq data, we utilized the "AUCell" method from the irGSEA package. The gene set files for GO Biological Processes (GOBP), HALLMARK, and REACTOME were obtained from The Molecular Signatures Database (MSigDB) (https://www.gsea-msigdb.org/gsea/msigdb) using the msigdbr package (Table S5). The signature genes of M2 macrophage polarization were derived from the supplementary materials of a previously published study by Azizi et al. [77] (Table S5).

### Single-cell metabolic activity analysis

To evaluate the metabolic pathway activity of meCAFs, we utilized the SCPA R package [78] to analyze meCAFs in the pan-cancer single-cell dataset, using the metabolic pathway gene sets obtained from the supplementary materials of Bibby et al.'s study [78].

Furthermore, we employed the scMetabolism R package [79] with default parameters to quantify the metabolic activity of four distinct subtypes of CAFs.

### Trajectory analysis

The slingshot R package [80] was used for inferring cell lineages and pseudotime. It utilized a clustering-based minimum spanning tree (MST) to identify the lineage structure and applies simultaneous principal curves to fit branch curves to these lineages. The getCurves function was employed to obtain smoothed trajectory curves.

Based on the inferred pseudotime, we utilized the GeneSwitches R package [81] to identify gene expression events within specific trajectory. The binarize_exp function was employed to convert the single-cell gene expression matrix into a binary state, using the following parameters: binarize_cutoff = 0.05 and fix_cutoff = TRUE. Subsequently, a logistic regression model was fitted and the switching time was estimated using the find_switch_logistic_fastglm function. Genes that satisfied the criteria (zero_pct = 0.9, r2cutoff = 0.02) were selected as switch genes. To determine the switch pathways, genes were initially filtered based on the following parameters: zero_pct = 0.9 and r2cutoff = 0.1. Finally, the find_switch_pathway function (sig_FDR = 0.05, pathways = msigdb_h_c2_c5) was employed, utilizing a hypergeometric test, to extract the switch pathways.

### Transcriptional homogeneity analysis

In order to estimate the heterogeneity of different CAFs subpopulations, we performed transcriptional homogeneity analysis on CAFs in the pan-cancer scRNA-seq dataset, adopting the computational approach described by Marjanovic et al. [82]. Specifically, using the top 100 marker genes of each cluster found by the FindAllMarkers function of the Seurat R package, we discretized expression per gene into 10 bins. Then, we subsampled 100 cells for each tumor sample 100 times and calculated the median value of the pairwise normalized mutual information (NMI). NMI between each pair of cells x and y was calculated according to the following 3 steps: (1) mutual information (MI) $I\left(X;Y\right)={\sum }_{x}{\sum }_{y}p(x,y)\mathrm{log}\frac{p(x,y)}{p\left(x\right)p(y)}$; (2) entropy of each cell $H\left(X\right)=\sum_{x}p(x)\mathrm{log}(p\left(x\right))$; (3) $NMI\left(X,Y\right)=\frac{I(X;Y)}{\sqrt{H\left(X\right)H(Y)}}$

### Spatial trajectory analysis

To investigate the dynamic biological processes occurring between high-density regions of iCAFs and mCAFs within the spatial context, we utilized the SPATA2 R package. Firstly, we transformed the Seurat object into a Spata object using the transformSeuratToSpata function. Then, employing the createTrajectories function, we generated a spatial trajectory starting from the high-density region of iCAFs and ending at the high-density region of mCAFs. The plotTrajectoryFeaturesDiscrete function was used to visualize the changes in cell proportions along the trajectory. Additionally, the plotTrajectoryGeneSets function was utilized to depict the variations in gene sets along the trajectory.

### Cell–cell interaction analysis

To infer cell–cell interaction within the spatial context, we utilized the SpaTalk R package [83]. Firstly, we created a SpaTalk object using the createSpaTalk function. Subsequently, the dec_cci function was applied with default parameters to identify ligand-receptor pairs involved in the interaction between CAFs and TME. The ligand-receptor pairs for each tissue slice were ranked based on their occurrence frequency, and the results from all tissue slices were integrated using the RRA algorithm [27]. To visualize the inferred intracellular signaling pathways, we employed the plot_path2gene function.

We conducted comparative analysis of cell communication between iCAFs and immune cells in a cohort of BRCA patients receiving anti-PD-1 immunotherapy using the CellChat R package [84]. The netAnalysis_signalingChanges_scatter function and netVisual_bubble function were utilized to visualize the changes in signaling pathways and ligand-receptor pairs from pre-treatment to on-treatment in BRCA patients.

### Melanoma immunotherapy dataset collection

We collected the expression matrix and clinical information of the GSE91061 dataset [85] (referred to as the Riaz cohort) from the Gene Expression Omnibus (GEO) database (http://www.ncbi.nlm.nih.gov/geo/). FPKM normalized gene expression data was converted into log2 (TPM + 1) data. Furthermore, we obtained the expression matrix and clinical information of the Gide cohort [86] and Nathanson cohort [87] from the Tumor Immune Dysfunction and Exclusion (TIDE) database (http://tide.dfci.harvard.edu/) [88]. We performed batch effect correction using the "ComBat" function in the SVA R package.

### TCGA RNA-seq data processing

RNA-seq data and clinical profiles of BRCA, CRC, LIHC, OVCA, PRAD, UCEC, and skin cutaneous melanoma (SKCM) from the TCGA database were downloaded from GDC API. Count data was converted into log2 (TPM + 1) data.

### Survival analysis

Kaplan–Meier survival analyses were performed with survival R package and survminer R package. The cut-off value of continuous variables in the survival data was determined by the surv_cutpoint function of survminer R package. *P*-values were calculated by log-rank test.

### Immune score

The estimate R package [89] was utilized to calculate the Immune_score (estimate). The Immune_score (Roh) was determined as the geometric mean of gene expression levels of cytolytic markers, HLA molecules, IFN-γ pathway genes, chemokines, and adhesion molecules [55]. Cytolytic activity (CYT) was calculated as the geometric mean of GZMA and PRF1 [56]. Tertiary lymphoid structures (TLS) were determined based on the mean expression of TLS-signature genes [57]. IFNy and expanded immune scores were obtained by averaging the gene expression levels of the included genes for the IFN-γ (6-gene) and expanded immune (18-gene) signatures, respectively [58]. Lastly, the Tcell inflamed score was calculated as the weighted sum of Tcell inflamed signature genes after housekeeping normalization [58]. The detailed information is provided in Table S11.

### Mutation, CNV, and DNA methylation analysis

We utilized the TCGAbiolinks R package [90] to download somatic mutation data and CNV data from the TCGA database for melanoma patients. The maftools R package [91] was employed for analyzing and visualizing the somatic mutation data. The TMB was calculated using the tmb function. Fisher's exact test was conducted to identify mutation genes with differential frequencies between groups with high and low iCAF scores. For the CNV data, the GISTIC2.0 [92] analysis module available on the GenePattern (https://cloud.genepattern.org) [93] was used to detect significantly amplified or deleted genomic regions. The burden of copy number alterations was quantified by counting the total number of genes exhibiting copy number gains or losses at both the focal and arm levels. The DNA methylation data and CNV data for melanoma patients, obtained from the UCSC Xena database (https://xenabrowser.net/datapages/), were employed for the analysis of immune modulators.

### Estimation of immune cell infiltration levels

We obtained gene signatures of 28 tumor-infiltrating lymphocytes (TILs) from the TISIDB database (http://cis.hku.hk/TISIDB) [94]. Subsequently, we employed the ssGSEA algorithm from the GSVA R package [95] to estimate the immune cell enrichment scores for each tumor sample.

### PROGENy analysis

The progeny R package [96] was utilized to infer the activity of 14 cancer-related pathways using default parameters.

### Immunofluorescence staining

The tissue microarrays for BRCA and LIHC were purchased from Shanghai Qutdo Biotech Company (Shanghai, China). For the immunofluorescence staining aimed at validating the existence of CAF subtypes, we followed the protocol outlined below. Immunofluorescence staining was performed using the Quadruple-Fluorescence immunohistochemical mouse/rabbit kit (Immunoway) to detect the expression of specific markers. The microarray was placed on a slide warmer and baked at 60 °C for 60 min to ensure adhesion. A two-in-one dewaxing and antigen retrieval reagent was added to a retrieval box and heated to boiling. Subsequently, the microarray was immersed in the boiling dewaxing and antigen retrieval reagent, ensuring complete submersion of the tissue. They were heated at medium flame for 30 min. The retrieval box was then removed from the heat source and allowed to naturally cool to room temperature. Following this, the microarray was transferred to a beaker containing distilled water and rinsed 5–6 times. Excess moisture around the tissue was blotted using filter paper. the microarray was then incubated with peroxidase-blocking buffer at room temperature for 15 min, followed by washing with PBS three times for 2 min each. Next, primary antibodies, including CENPF (Rabbit, 1:200, Immunoway), HILPDA (Rabbit, 1:200, Bioss), MMP-11 (Rabbit, 1:200, Immunoway), and CFD (Rabbit, 1:200, Immunoway), were diluted and applied to the microarray, ensuring complete coverage. Incubation was carried out at 37 °C for 1–2 h (or overnight at 4 °C in a humid chamber), followed by three washes with PBS for 2 min each. After blotting the excess moisture, the microarray was incubated with an HRP-conjugated anti-rabbit/mouse IgG secondary antibody at room temperature for 30 min. The sections were washed again with PBS three times for 2 min each. For fluorescence labeling, Tyramide working solution was added and incubated for 10 min. Subsequently, the sections were washed with PBS three times for 2 min each. the microarray was placed in a retrieval box, and an antibody stripping solution was added. Microwave heating was performed at high power for 3 min and at medium–low power for 15 min. After natural cooling, the sections were washed with PBS three times for 2 min each. Finally, DAPI staining solution was added and mounting medium was applied to cover the microarray, ensuring contact without trapping air bubbles. Subsequently, the sections were scanned and imaged using a digital slide scanner microscope.

For the immunofluorescence staining aimed at validating the spatial proximity of CFD-positive cells and CD8-positive cells, we followed the protocol outlined below. The microarray was placed on a slide warmer at 60 °C for 30 min. Subsequently, it was sequentially immersed in xylene (first and second), followed by various concentrations of ethanol and water, each for 5 min. Antigen retrieval was performed using trypsin at 37 °C for 20 min. The microarray was then washed three times with PBS buffer for 5 min each. Endogenous peroxidase activity was blocked by incubating the microarray with a peroxidase blocking agent at room temperature for 10 min, followed by three washes with PBS buffer. To block non-specific binding, goat serum was applied to the microarray at 37 °C for 15 min, after which the excess serum was removed. Primary antibodies (CD8 Ms 1:100, CFD Rb1:100) were added and left overnight at 4 °C. The microarray was then washed three times with PBS buffer, and secondary antibodies (Alexa Fluor 488@Ms, Alexa Fluor 594@Rb) were applied. The microarray was incubated at 37 °C for 30 min, and all subsequent steps were performed in a light-protected environment. After three washes with PBS buffer, DAPI staining solution was added, followed by three additional washes with PBS buffer. Finally, the microarray was mounted using a tissue mounting medium containing an anti-fading agent, ensuring the absence of bubbles. The imaging of the microarray slices was performed using a digital slide scanner microscope, capturing the desired observations.

### Supplementary Information

**Additional file 1:** **Table S1.** Sample information of public single cell RNA sequencing data.

**Additional file 2:** **Table S2.** Sample information of public spatial transcriptomic data.

**Additional file 3:** **Table S3.** Marker genes for each cell type.

**Additional file 4:** **Table S4.** The top differentially expressed genes in each CAF subtype.

**Additional file 5:** **Table S5.** Gene signatures for various CAF functions.

**Additional file 6:** **Table S6.** Sample information of newly generated single cell RNA sequencing data.

**Additional file 7:** **Table S7.** Regulon specificity scores for each CAF subtype.

**Additional file 8:** **Table S8.** Metabolic pathway enrichment analysis results of meCAFs using SCPA R Package.

**Additional file 9:** **Table S9.** Integrated ranking of LRIs from each CAF subtype to TME using RRA algorithm.

**Additional file 10:** **Table S10.** Ligands promoting M2 macrophage polarization.

**Additional file 11:** **Table S11.** Immune scores.

**Additional file 12:** **Figure S1.** Quality control of scRNA-seq and ST data a Numeric table of quality-controlled scRNA-seq and ST data b Boxplot showing number of UMIs per cell from different samples in scRNA-seq data c Boxplot showing number of genes per cell from different samples in scRNA-seq data d Boxplot showing number of UMIs per cell from different sections in ST data e Boxplot showing number of genes per cell from different sections in ST data.

**Additional file 13:** **Figure S2.** UMAP plots showing the major cell types in each scRNA-seq dataset of this pan-cancer analysis.

**Additional file 14:** **Figure S3.** UMAP plots showing the CopyKAT classification results in each scRNA-seq dataset of this pan-cancer analysis.

**Additional file 15:** **Figure S4.** UMAP plots showing the subsets of myeloid cells in each scRNA-seq dataset of this pan-cancer analysis.

**Additional file 16:** **Figure S5.** UMAP plots showing the subsets of T&NK in each scRNA-seq dataset of this pan-cancer analysis.

**Additional file 17:** **Figure S6.** UMAP plots showing the co-embedding results of scRNA-seq and ST data using CellTrek.

**Additional file 18:** **Figure S7.** Transcriptional heterogeneity among CAF subtypes. a Phylogenetic tree of major cell types across six cancer types. b Boxplot showing transcriptional homogeneity of different CAF subtypes quantified by NMI. Statistical analysis was performed using Wilcoxon rank-sum tests; *P< 0.05, **P< 0.01, ***P< 0.001.

**Additional file 19:** **Figure S8.** Boxplot showing the local inverse Simpson’s Index (LISI) of fibroblasts before and after batch correction. Statistical analysis was performed using Wilcoxon rank-sum tests; *P< 0.05, **P< 0.01, ***P< 0.001.

**Additional file 20:** **Figure S9.** Representative immunofluorescence images of CFD (deep yellow, iCAF), MMP11 (light yellow, mCAF), HILPDA (red, meCAF) and CENPF (green, pCAF) in tissues from patients with BRCA. Scale bar represents 20 μm.

**Additional file 21:** **Figure S10.** CAF heterogeneity in other cancer types. a UMAP plots showing the subsets of CAFs in NSCLC. b UMAP plots showing the subsets of CAFs in Melanoma. c UMAP plots showing the subsets of CAFs in HNSCC. d UMAP plots showing the subsets of CAFs in EMC. e UMAP plots showing the subsets of CAFs in MEC. f Volcano plot showing genes upregulated in fibroblasts derived from tumor tissues of HNSCC patients, compared to fibroblasts derived from adjacent non-tumor tissues. g Enriched GO functions of upregulated genes in fibroblasts derived from tumor tissues of HNSCC patients. h Volcano plot showing genes upregulated in fibroblasts derived from tumor tissues of MEC patients, compared to fibroblasts derived from adjacent non-tumor tissues. i Enriched GO functions of upregulated genes in fibroblasts derived from tumor tissues of MEC patients.

**Additional file 22:** **Figure S11.** Bubble heatmap showing metabolic pathway activities scored by scMetabolism in each CAF subtype.

**Additional file 23:** **Figure S12.** Spatial trajectory analysis of CAFs. a Spatial trajectory from high-density areas of iCAFs to high-density areas of mCAFs in OVCA1. B Changes in cell proportions of CAF subtypes along the trajectory direction in OVCA1. C Changes in pathway activity of CAF subtypes along the trajectory direction in OVCA1. D Spatial trajectory from high-density areas of iCAFs to high-density areas of mCAFs in CRC1. E Changes in cell proportions of CAF subtypes along the trajectory direction in CRC1. F Changes in pathway activity of CAF subtypes along the trajectory direction in CRC1.

**Additional file 24:** **Figure S13.** Integrated ranking of various functional LRIs based on number of LRIs from mCAFs to endothelial cells using RRA algorithm across 22 tissue slices.

**Additional file 25:** **Figure S14.** Integrated ranking of various functional LRIs based on number of LRIs from iCAFs to macrophages using RRA algorithm across 22 tissue slices.

**Additional file 26:** **Figure S15.** Integrated ranking of various functional LRIs based on number of LRIs from iCAFs to CD8+ T cells using RRA algorithm across 22 tissue slices.

**Additional file 27:** **Figure S16.** Effect of iCAFs on immune cells through paracrine signaling. A GO enrichmet of ligands from iCAFs to B cells. b GO enrichmet of ligands from iCAFs to dendritic cells. c GO enrichmet of ligands from iCAFs to mast cells. d GO enrichmet of ligands from iCAFs to neutrophils. E GO enrichmet of ligands from iCAFs to NK cells. f GO enrichmet of ligands from iCAFs to Tregs. G Integrated ranking of LRIs based on number of LRIs from iCAFs to B cells using RRA algorithm across 22 tissue slices. h Integrated ranking of LRIs based on number of LRIs from iCAFs to dendritic cells using RRA algorithm across 22 tissue slices. i Integrated ranking of LRIs based on number of LRIs from iCAFs to mast cells using RRA algorithm across 22 tissue slices. j Integrated ranking of LRIs based on number of LRIs from iCAFs to neutrophils using RRA algorithm across 22 tissue slices. k Integrated ranking of LRIs based on number of LRIs from iCAFs to NK cells using RRA algorithm across 22 tissue slices. l Integrated ranking of LRIs based on number of LRIs from iCAFs to Tregs using RRA algorithm across 22 tissue slices.

**Additional file 28:** **Figure S17.** Spearman correlation analysis of LGALS1 and NFATC2 with PDCD1. A Spearman correlation analysis of LGALS1 with PDCD1. B Spearman correlation analysis of NFATC2 with PDCD1.

**Additional file 29:** **Figure S18.** Downstream analysis of the interaction between iCAFs and CD8+ T cells. a Intracellular signaling network triggered by LGALS1- PTPRC interaction. B Slingshot trajectory analysis of the 13 clusters of CD8+ T cells. c Pseudotime of the 6 lineages of CD8+ T cells calculated by Slingshot. D Bubble heatmap showing the expression of marker genes for CD8+ T cell subtypes in pan-cancer. E GeneSwitches analysis of switching genes in Lineage 1.

**Additional file 30:** **Figure S19.** Anti-PD1 treatment influences transcriptional characteristics of iCAFs. A Boxplot showing the differences in cell proportions between patients with clonal expansion before and during anti-PD-1 treatment. Statistical analysis was performed using unpaired t-tests; *P< 0.05, **P< 0.01, ***P< 0.001. b Boxplot showing the differences in cell proportions between patients without clonal expansion before and during anti-PD-1 treatment. Statistical analysis was performed using unpaired t-tests; *P< 0.05, **P< 0.01, ***P< 0.001. c Volcano plot showing DEGs of iCAFs between pre- and on-anti-PD-1 treatment. d GO enrichmet of DEGs of iCAFs between pre- and on-anti-PD-1 treatment. e Density heatmaps showing the comparison of pathway activities of iCAFs scored by AUCell between pre- and on-anti-PD-1 treatment. Statistical analysis was performed using Wilcoxon rank-sum tests; *P< 0.05, **P< 0.01, ***P< 0.001. f UMAP plot showing the myeloid cells subpopulations in BRCA immunotherapy cohort. g Bubble heatmap showing the expression of marker genes for myeloid cells subpopulations in BRCA immunotherapy cohort. h UMAP plot showing the T cells subpopulations in BRCA immunotherapy cohort. i Bubble heatmap showing the expression of marker genes for T cells subpopulations in BRCA immunotherapy cohort.

**Additional file 31:** **Figure S20.** The immune and genomic landscape of melanoma patients based on high and low iCAF scores. a Boxplot showing the comparison of iCAF scores in anti−PD1 therapy responders and non-responders, as well as anti−CTLA−4 therapy responders and non-responders, among melanoma patients. Statistical analysis was performed using Wilcoxon rank-sum tests; *P< 0.05, **P< 0.01, ***P< 0.001. b Mutation landscapes of melanoma patients with high and low iCAF scores. Statistical analysis was performed using Fisher's exact test; *P< 0.05, **P< 0.01, ***P< 0.001. c Boxplot showing the comparison of TMB in melanoma patients with high and low iCAF score. Statistical analysis was performed using Wilcoxon rank-sum tests; *P< 0.05, **P< 0.01, ***P< 0.001. d Boxplot showing the comparison of copy number gain burden or copy number loss burden at arm-level or focal-level in melanoma patients with high and low iCAF score. Statistical analysis was performed using Wilcoxon rank-sum tests; *P< 0.05, **P< 0.01, ***P< 0.001. e Boxplot showing the comparison of immune modulators expression levels in melanoma patients with high and low iCAF score. Statistical analysis was performed using Wilcoxon rank-sum tests; *P< 0.05, **P< 0.01, ***P< 0.001. f Boxplot showing the comparison of pathway activities scored by PROGENy in melanoma patients with high and low iCAF score. Statistical analysis was performed using Wilcoxon rank-sum tests; *P< 0.05, **P< 0.01, ***P< 0.001. g Heatmap showing immune cell infiltration levels in melanoma patients based on high and low iCAF scores.

## Data Availability

The raw sequence data reported in this paper have been deposited in the Genome Sequence Archive in National Genomics Data Center, China National Center for Bioinformation / Beijing Institute of Genomics, Chinese Academy of Sciences (GSA-Human: HRA004998) that are publicly accessible at https://ngdc.cncb.ac.cn/gsa-human. The integrated ST data from 22 tissue slices and their corresponding scRNA-seq data have been deposited in Synapse under the accession code syn51758773 (https://www.synapse.org/#!Synapse:syn51758773/). The analysis and visualization of CAFs in pan-cancer can be performed at https://chenxisd.shinyapps.io/pancaf/. Reanalyzed publicly available scRNA-seq data can be accessed from the GEO database under accession codes: GSE176078 [97], GSE166555 [98], GSE149614 [99], GSE184880 [100], GSE203612 [101], GSE207422 [18], GSE215120 [19], GSE181919 [7]. The scRNA-seq data of PRAD from Chen et al. were downloaded from http://www.pradcellatlas.com/. The scRNA-seq data of BRCA patients receiving pembrolizumab from Bassez et al. were downloaded from https://lambrechtslab.sites.vib.be/en/single-cell [41]. Reanalyzed publicly available ST data can be accessed from the GEO database under accession codes: GSE176078 [97], GSE203612 [101]. The ST data of CRC from Wu et al. were downloaded from http://www.cancerdiversity.asia/scCRLM/ [79]. The ST data for LIHC1, LIHC2, LIHC3, and LIHC4 from Wu et al. were downloaded from http://lifeome.net/supp/livercancer-st/data.htm [102]. The ST data for OVCA_10x were downloaded from https://www.10xgenomics.com/resources/datasets/human-ovarian-cancer-1-standard. The ST data for PRAD1 were downloaded from https://www.10xgenomics.com/resources/datasets/human-prostate-cancer-acinar-cell-carcinoma-ffpe-1-standard. The ST data for PRAD2 were downloaded from https://www.10xgenomics.com/resources/datasets/human-prostate-cancer-adenocarcinoma-with-invasive-carcinoma-ffpe-1-standard-1-3-0. Reanalyzed publicly available RNA-seq data of melanoma patients undergoing immunotherapy (Riaz cohort) can be accessed from the GEO database under accession code GSE91061 [85]. Reanalyzed publicly available RNA-seq data of melanoma patients undergoing immunotherapy (Gide cohort [86] and Nathanson cohort [87]) were downloaded from TIDE database (http://tide.dfci.harvard.edu/) [88].

## Abbreviations

CAF: Cancer-associated fibroblast
TME: Tumor microenvironment
scRNA-seq: Single-cell RNA sequencing
EMC: Epithelial-myoepithelial carcinoma
MEC: Mucoepidermoid carcinoma
mCAF: Matrix cancer-associated fibroblast
iCAF: Inflammatory cancer-associated fibroblast
BRCA: Breast cancer
EMT: Epithelial-mesenchymal transition
ECM: Extracellular matrix
BC: Bladder carcinoma
HNSCC: Head and neck squamous cell carcinoma
PTC: Papillary thyroid carcinoma
LC: Lung cancer
ST: Spatial transcriptomics
CRC: Colorectal cancer
LIHC: Liver hepatocellular carcinoma
OVCA: Ovarian cancer
PRAD: Prostate adenocarcinoma
UCEC: Uterine corpus endometrial carcinoma
UMI: Unique molecular identifier
CNV: Copy number variation
SMC: Smooth muscle cell
meCAF: Metabolic cancer-associated fibroblast
PDAC: Pancreatic ductal adenocarcinoma
pCAF: Proliferative cancer-associated fibroblast
NSCLC: Non-small cell lung cancer
RRA: Robust rank aggregation
LRI: Ligand-receptor interaction
TCGA: The Cancer Genome Atlas
TF: Transcription factor
DEG: Differentially expressed gene
ICB: Immune checkpoint blockade
OS: Overall survival
PFS: Progression-free survival
TMB: Tumor mutational burden
CYT: Cytolytic activity
TLS: Tertiary lymphoid structures signature
PFT: Pericyte–fibroblast transition
GEM: Gel beads in emulsion
OR: Odds ratio
RAS: Regulon activity score
RSS: Regulon specificity score
MSigDB: Molecular Signatures Database
MST: Minimum spanning tree
GEO: Gene Expression Omnibus
SKCM: Skin cutaneous melanoma
LISI: Local inverse Simpson’s Index
