# Supplementary figures and images for: Pan-cancer spatially resolved single-cell analysis reveals the crosstalk between cancer-associated fibroblasts and tumor microenvironment
Source: Mol Cancer. 2023 Oct 13;22:170. doi: 10.1186/s12943-023-01876-x (PMC10571470; doi:10.1186/s12943-023-01876-x)

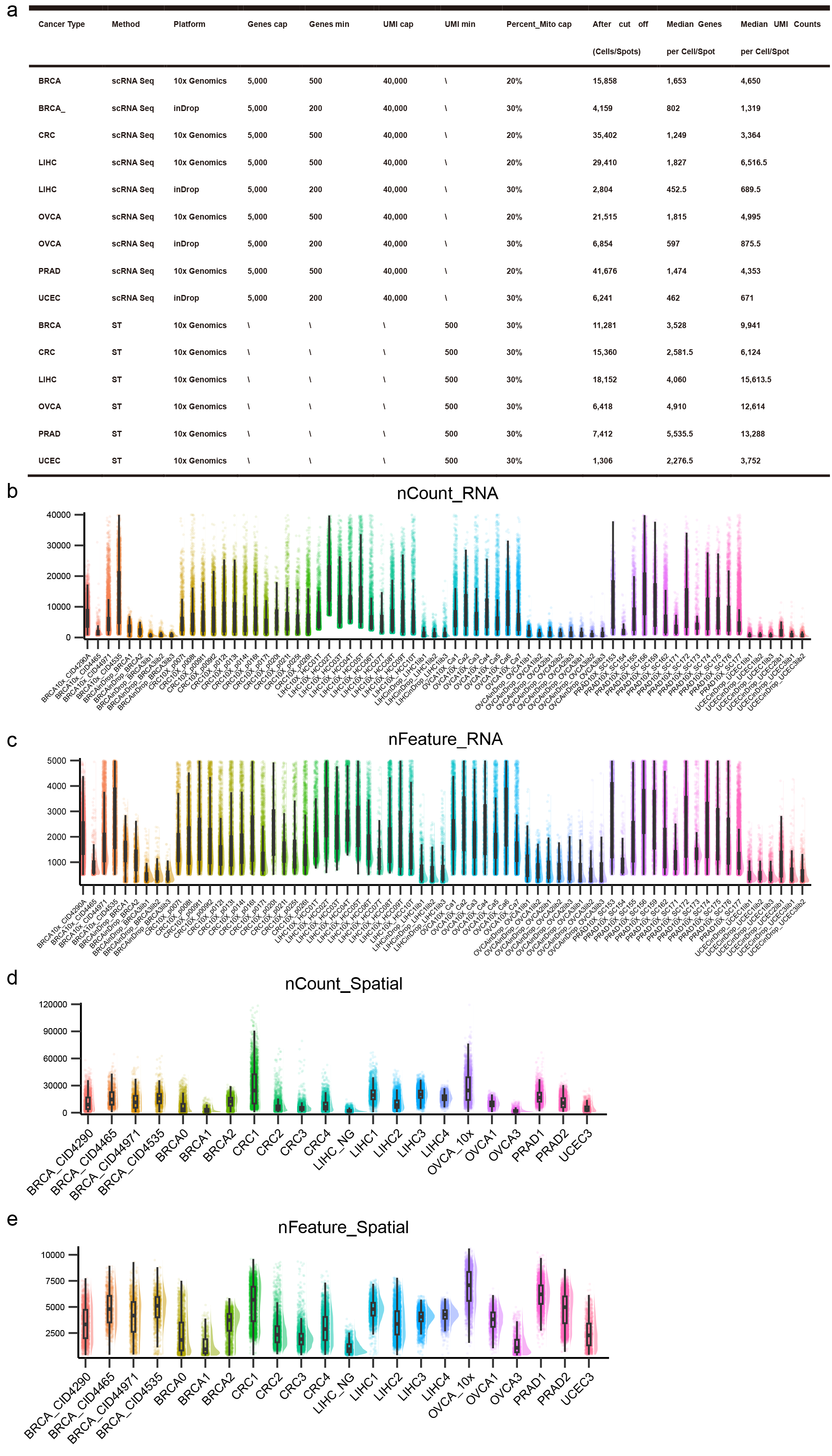

Supplement: Supplementary file 12 — Additional file 12: Figure S1. Quality control of scRNA-seq and ST data a Numeric table of quality-controlled scRNA-seq and ST data b Boxplot showing number of UMIs per cell from different samples in scRNA-seq data c Boxplot showing number of genes per cell from different samples in scRNA-seq data d Boxplot showing number of UMIs per cell from different sections in ST data e Boxplot showing number of genes per cell from different sections in ST data. [file 12943_2023_1876_MOESM12_ESM.tif]

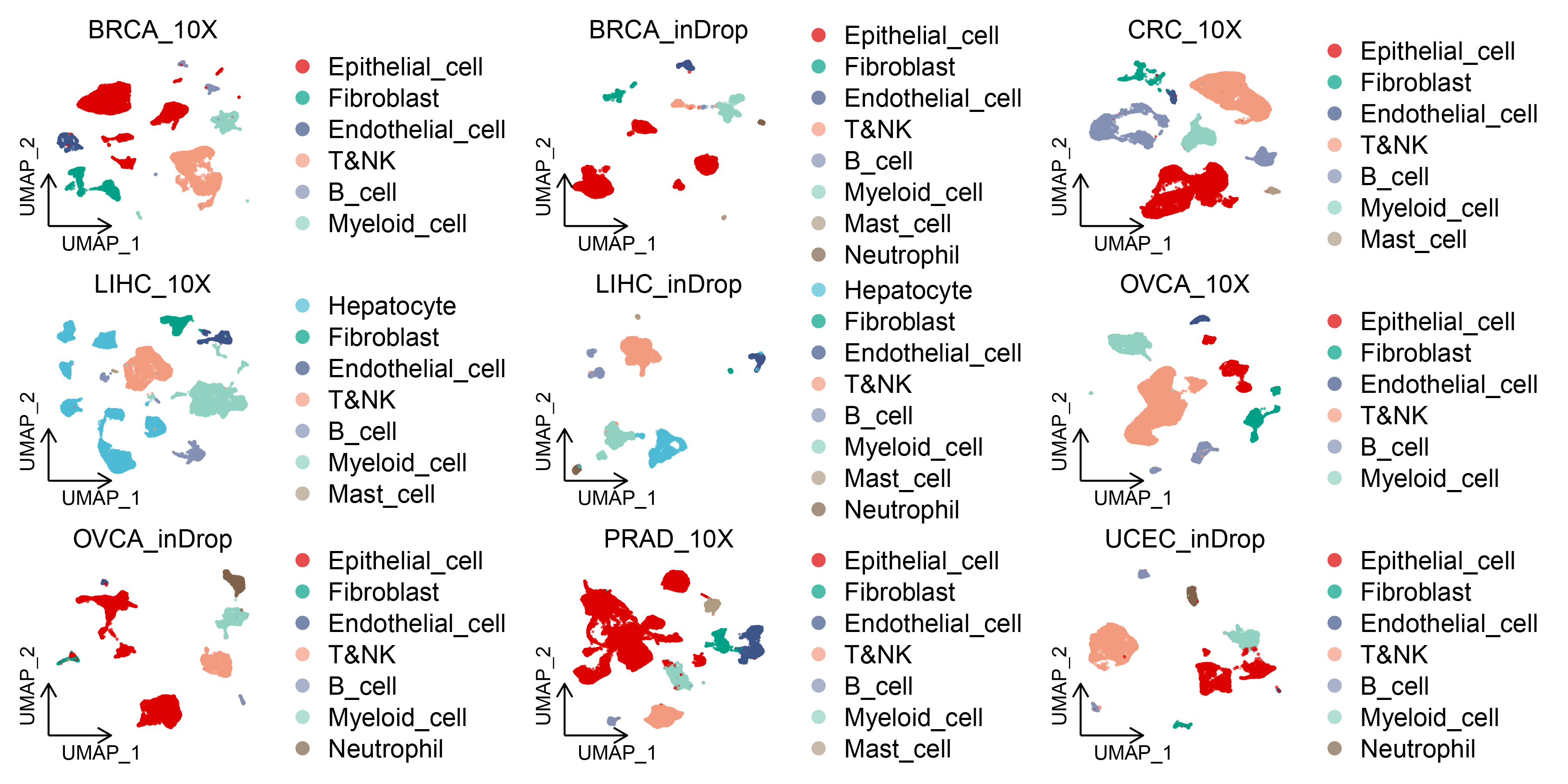

Supplement: Supplementary file 13 — Additional file 13: Figure S2. UMAP plots showing the major cell types in each scRNA-seq dataset of this pan-cancer analysis. [file 12943_2023_1876_MOESM13_ESM.tif]

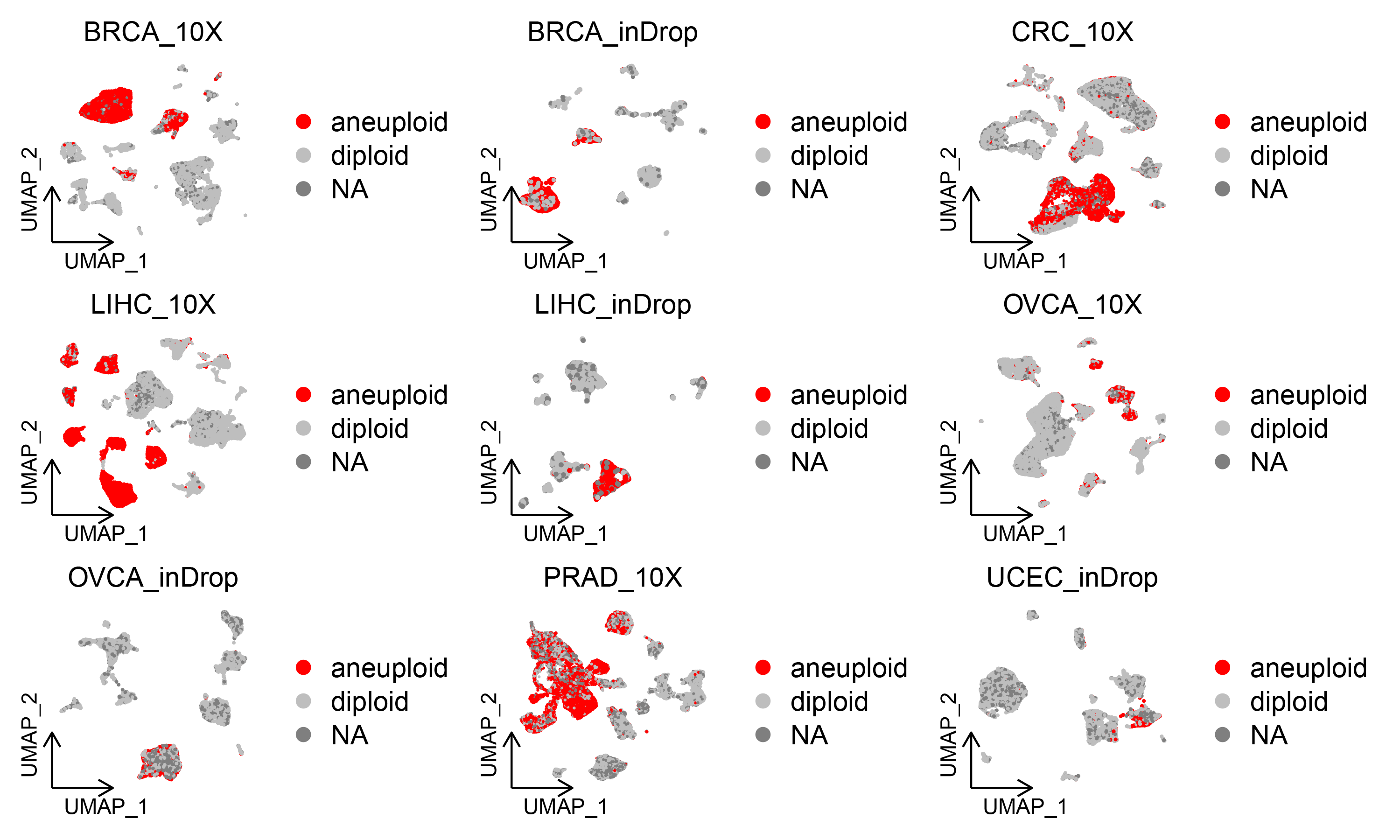

Supplement: Supplementary file 14 — Additional file 14: Figure S3. UMAP plots showing the CopyKAT classification results in each scRNA-seq dataset of this pan-cancer analysis. [file 12943_2023_1876_MOESM14_ESM.tif]

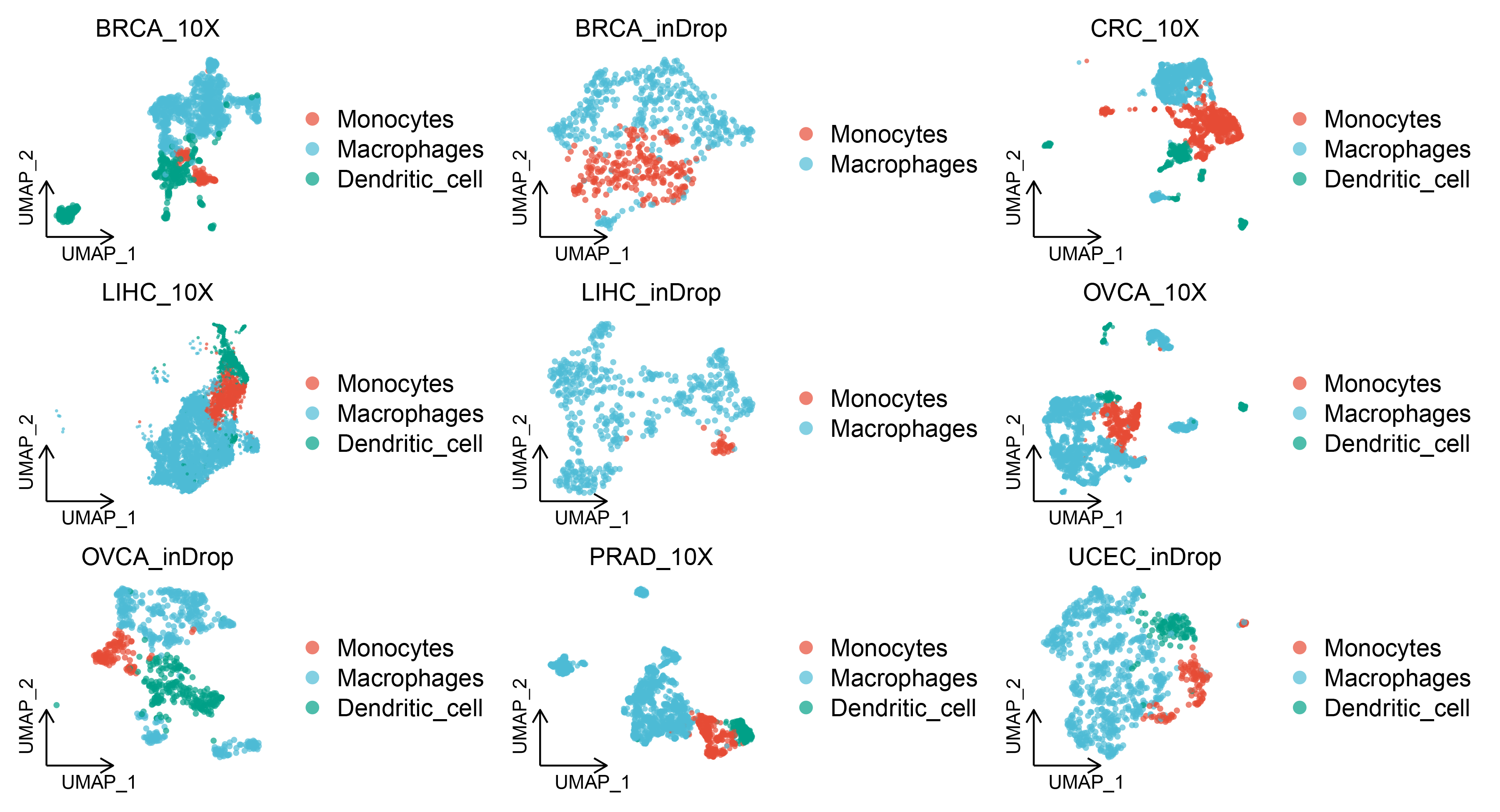

Supplement: Supplementary file 15 — Additional file 15: Figure S4. UMAP plots showing the subsets of myeloid cells in each scRNA-seq dataset of this pan-cancer analysis. [file 12943_2023_1876_MOESM15_ESM.tif]

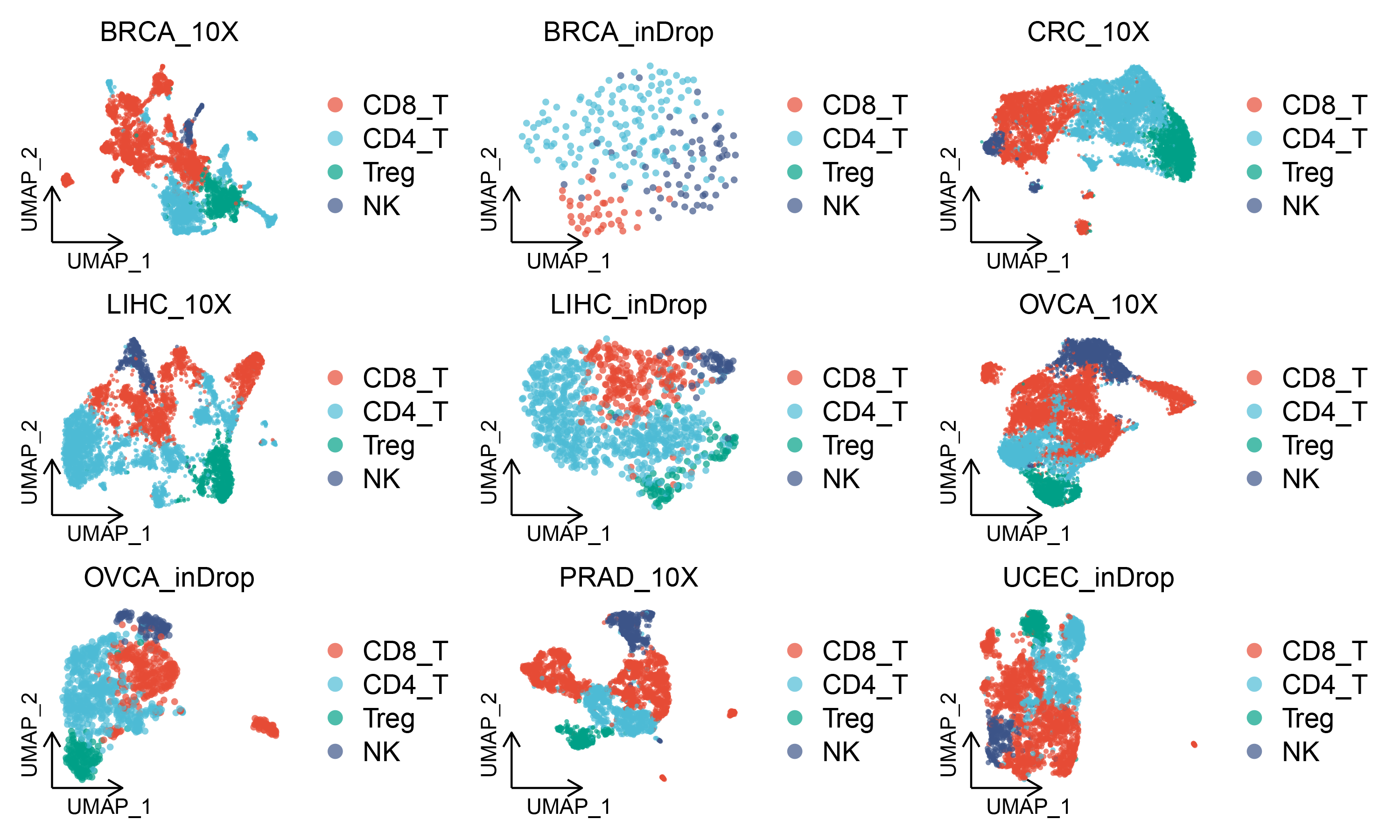

Supplement: Supplementary file 16 — Additional file 16: Figure S5. UMAP plots showing the subsets of T&NK in each scRNA-seq dataset of this pan-cancer analysis. [file 12943_2023_1876_MOESM16_ESM.tif]

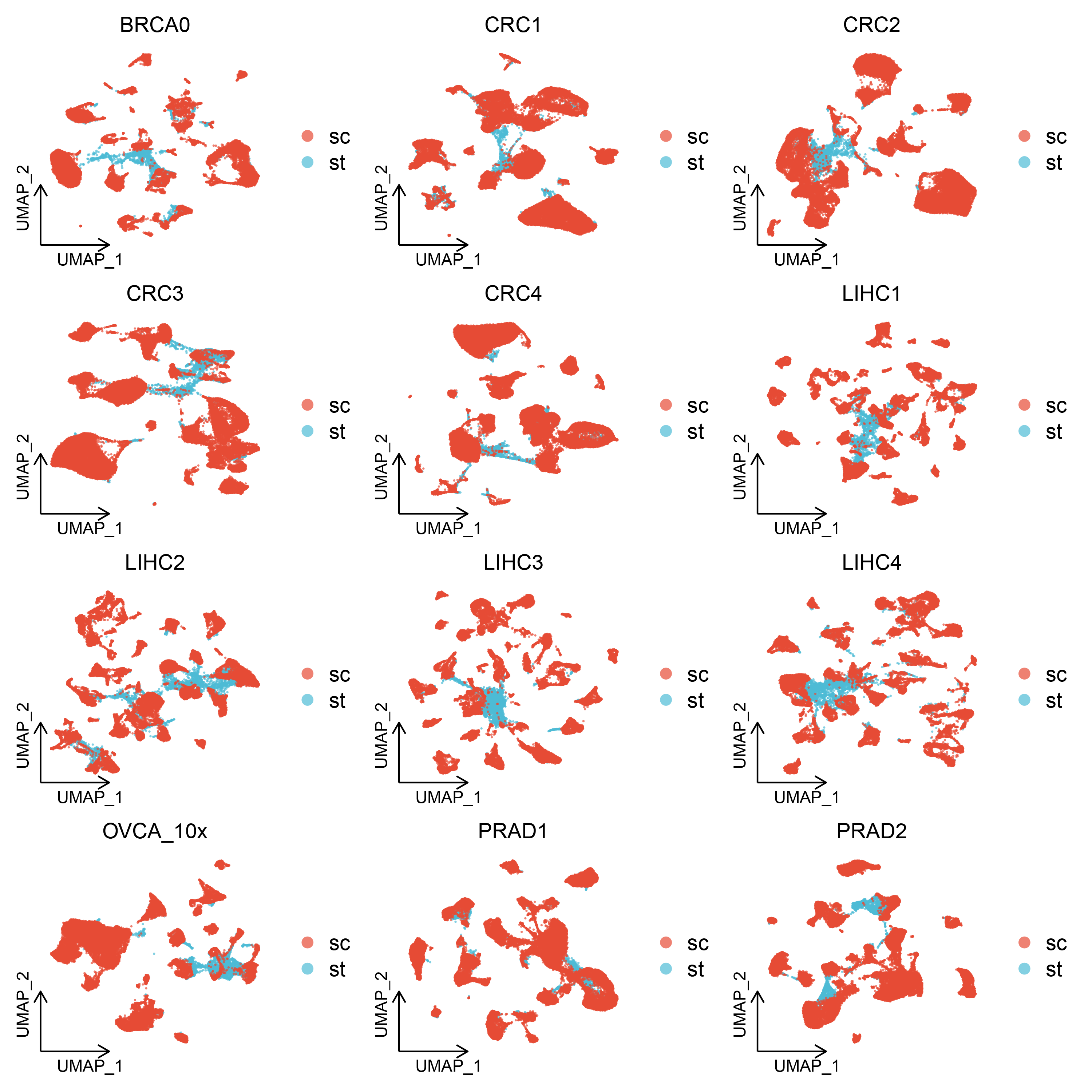

Supplement: Supplementary file 17 — Additional file 17: Figure S6. UMAP plots showing the co-embedding results of scRNA-seq and ST data using CellTrek. [file 12943_2023_1876_MOESM17_ESM.tif]

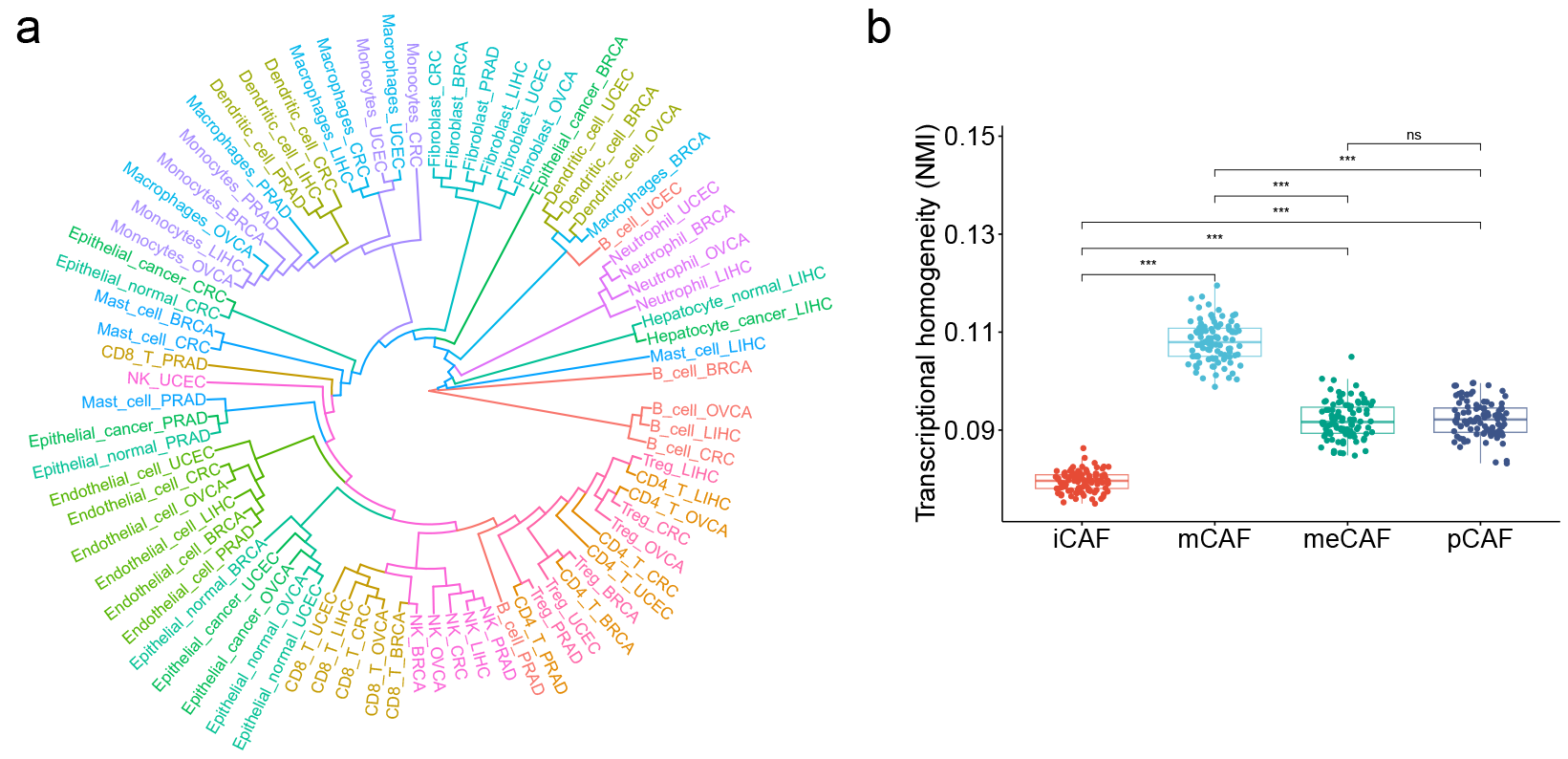

Supplement: Supplementary file 18 — Additional file 18: Figure S7. Transcriptional heterogeneity among CAF subtypes. a Phylogenetic tree of major cell types across six cancer types. b Boxplot showing transcriptional homogeneity of different CAF subtypes quantified by NMI. Statistical analysis was performed using Wilcoxon rank-sum tests; *P< 0.05, **P< 0.01, ***P< 0.001. [file 12943_2023_1876_MOESM18_ESM.tif]

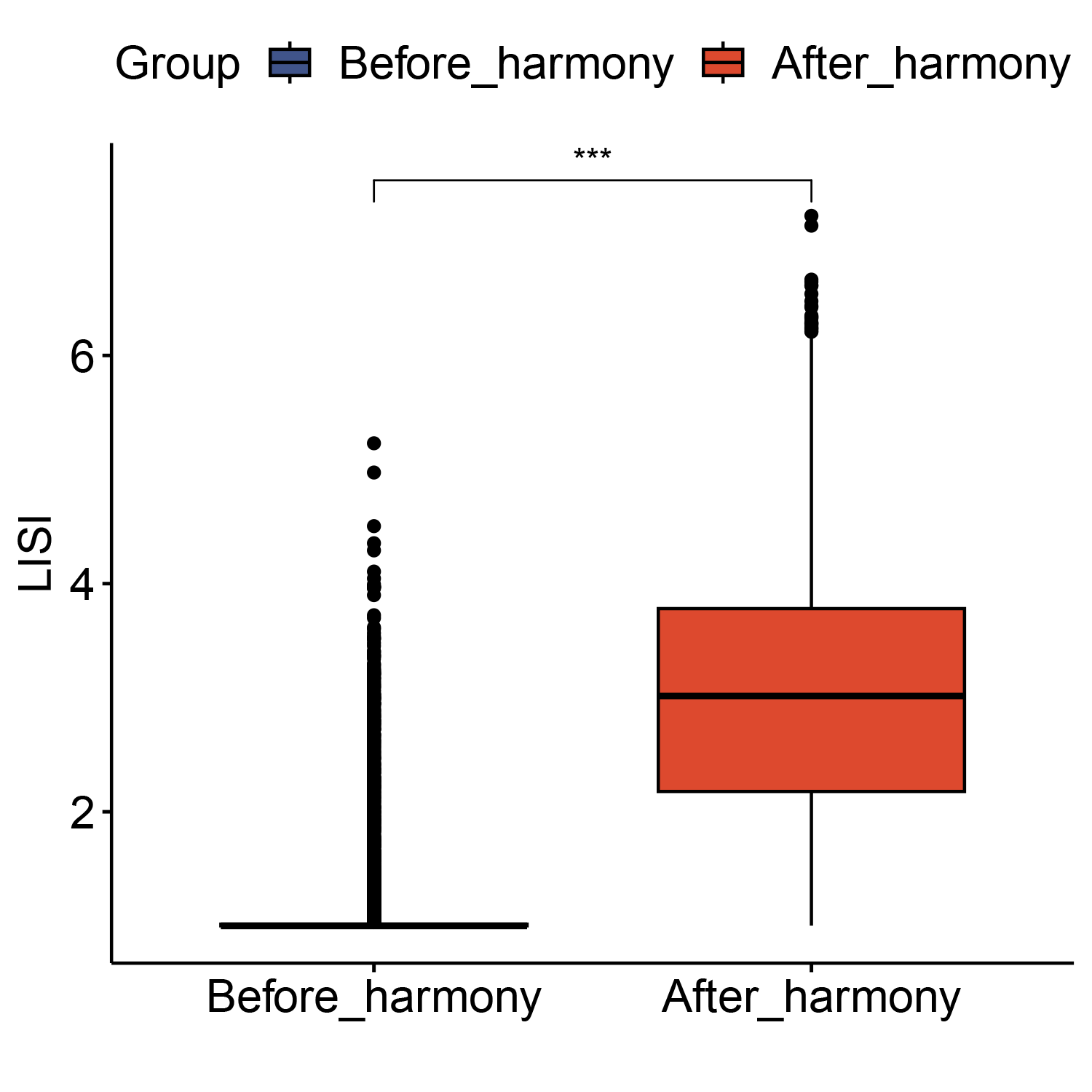

Supplement: Supplementary file 19 — Additional file 19: Figure S8. Boxplot showing the local inverse Simpson’s Index (LISI) of fibroblasts before and after batch correction. Statistical analysis was performed using Wilcoxon rank-sum tests; *P< 0.05, **P< 0.01, ***P< 0.001. [file 12943_2023_1876_MOESM19_ESM.tif]

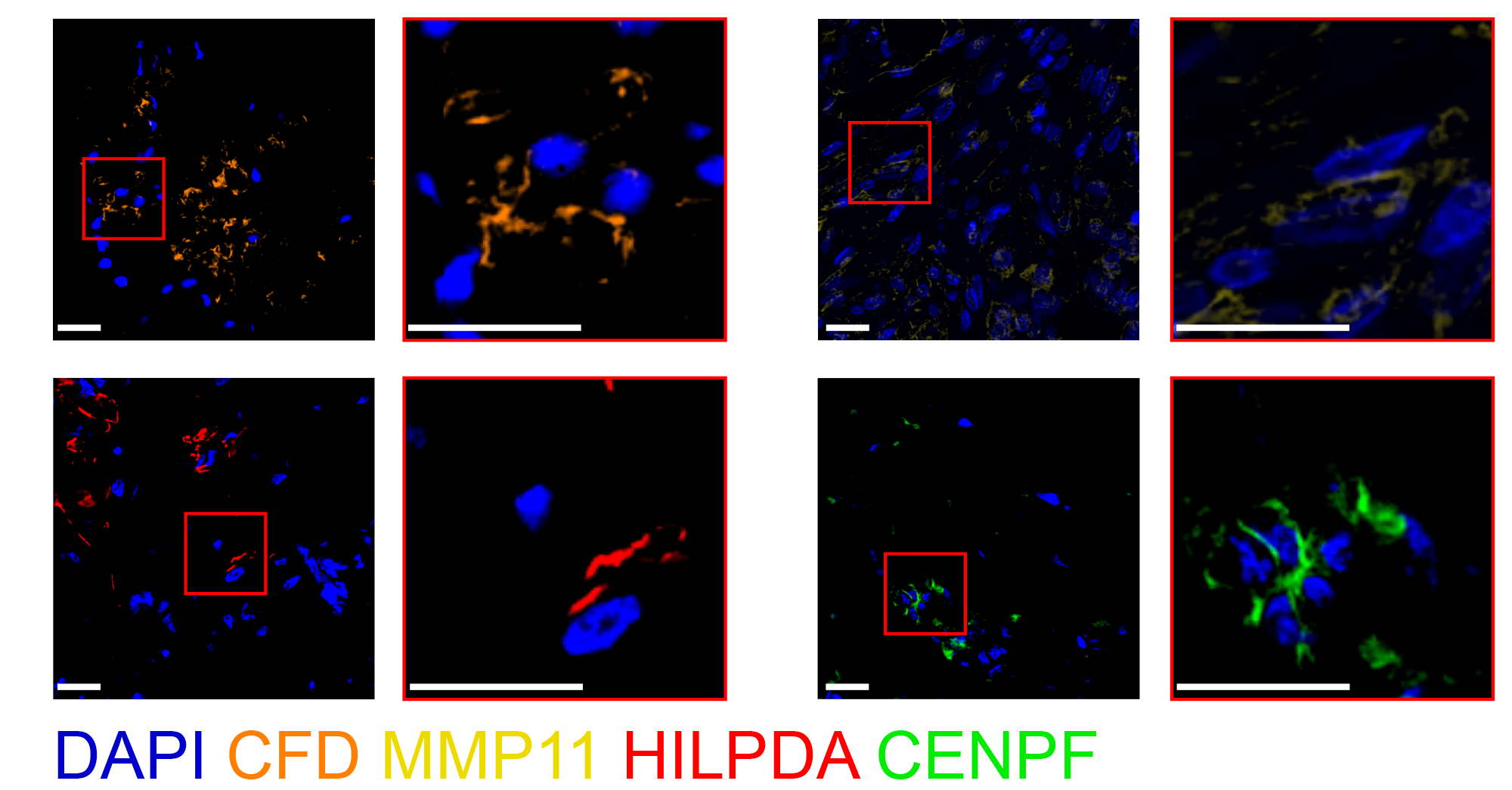

Supplement: Supplementary file 20 — Additional file 20: Figure S9. Representative immunofluorescence images of CFD (deep yellow, iCAF), MMP11 (light yellow, mCAF), HILPDA (red, meCAF) and CENPF (green, pCAF) in tissues from patients with BRCA. Scale bar represents 20 μm. [file 12943_2023_1876_MOESM20_ESM.tif]

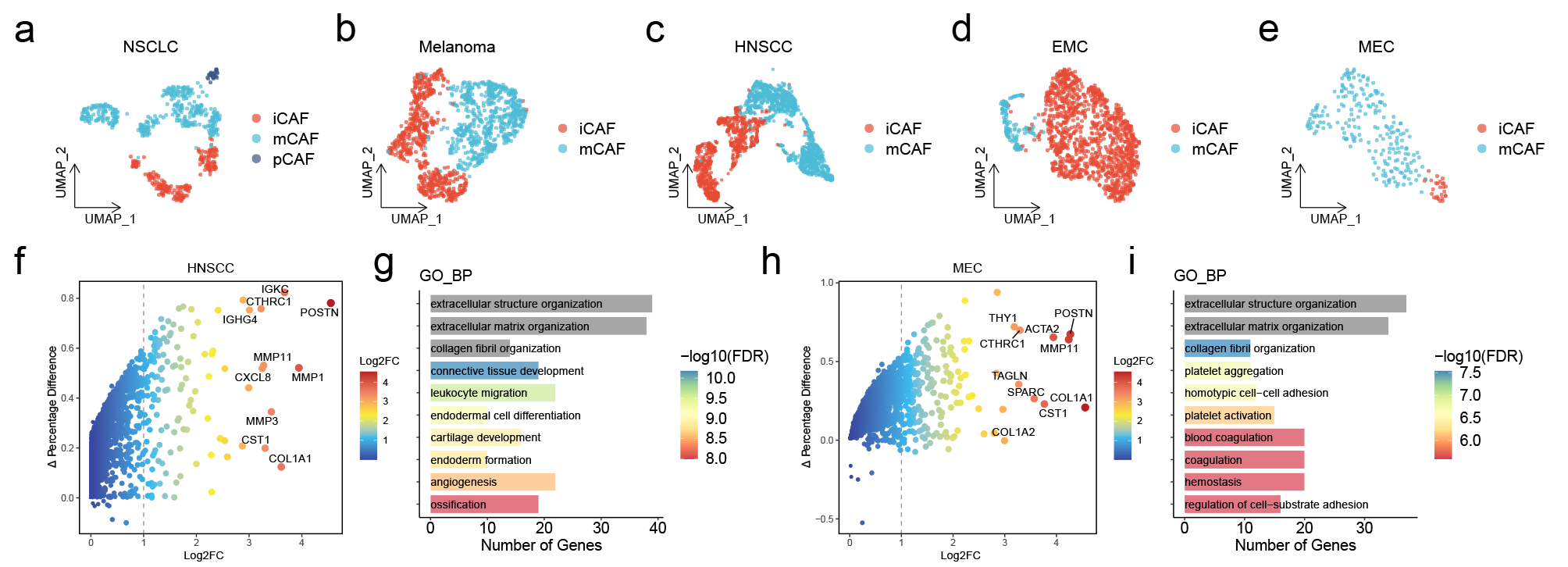

Supplement: Supplementary file 21 — Additional file 21: Figure S10. CAF heterogeneity in other cancer types. a UMAP plots showing the subsets of CAFs in NSCLC. b UMAP plots showing the subsets of CAFs in Melanoma. c UMAP plots showing the subsets of CAFs in HNSCC. d UMAP plots showing the subsets of CAFs in EMC. e UMAP plots showing the subsets of CAFs in MEC. f Volcano plot showing genes upregulated in fibroblasts derived from tumor tissues of HNSCC patients, compared to fibroblasts derived from adjacent non-tumor tissues. g Enriched GO functions of upregulated genes in fibroblasts derived from tumor tissues of HNSCC patients. h Volcano plot showing genes upregulated in fibroblasts derived from tumor tissues of MEC patients, compared to fibroblasts derived from adjacent non-tumor tissues. i Enriched GO functions of upregulated genes in fibroblasts derived from tumor tissues of MEC patients. [file 12943_2023_1876_MOESM21_ESM.tif]

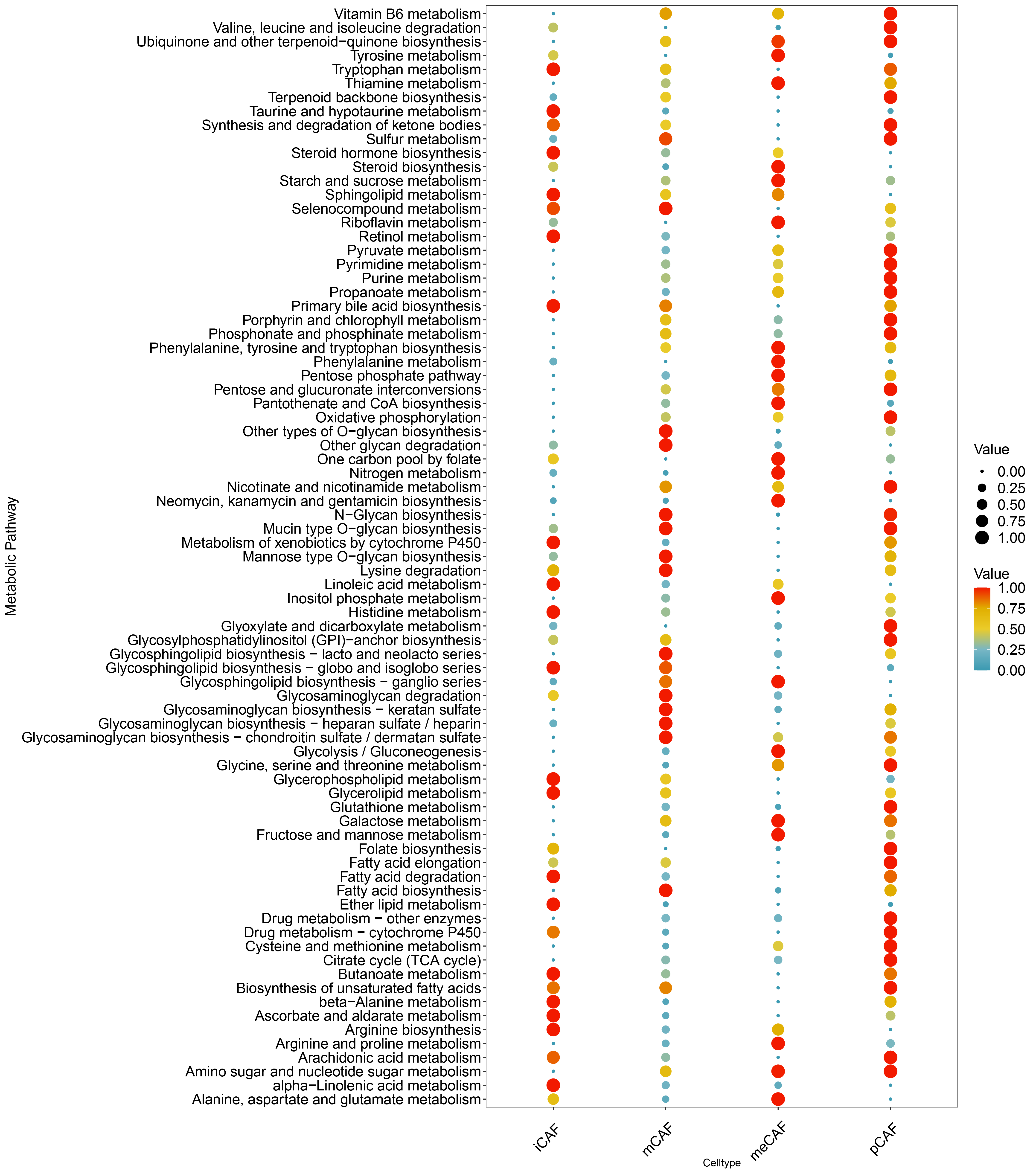

Supplement: Supplementary file 22 — Additional file 22: Figure S11. Bubble heatmap showing metabolic pathway activities scored by scMetabolism in each CAF subtype. [file 12943_2023_1876_MOESM22_ESM.tif]

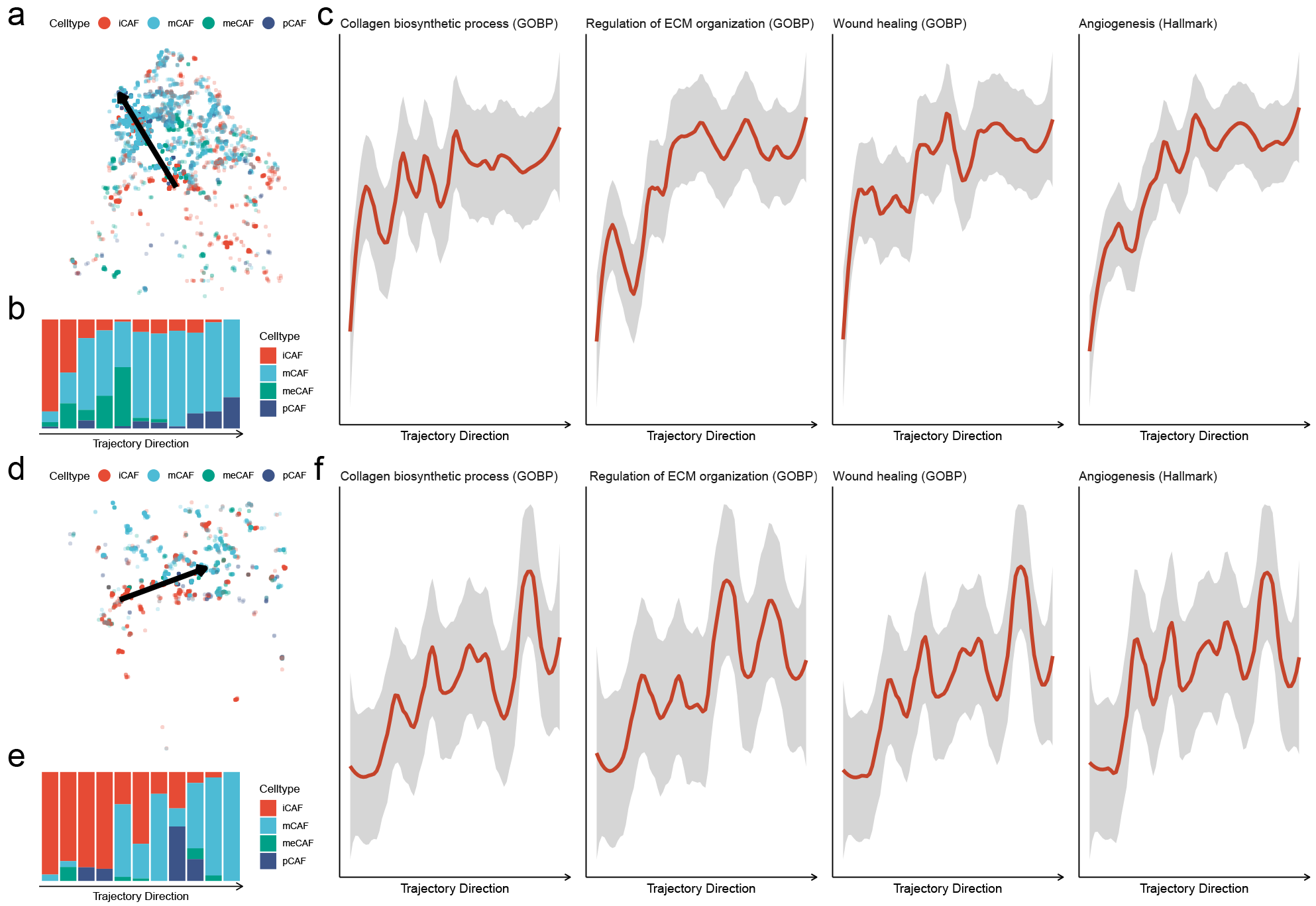

Supplement: Supplementary file 23 — Additional file 23: Figure S12. Spatial trajectory analysis of CAFs. a Spatial trajectory from high-density areas of iCAFs to high-density areas of mCAFs in OVCA1. B Changes in cell proportions of CAF subtypes along the trajectory direction in OVCA1. C Changes in pathway activity of CAF subtypes along the trajectory direction in OVCA1. D Spatial trajectory from high-density areas of iCAFs to high-density areas of mCAFs in CRC1. E Changes in cell proportions of CAF subtypes along the trajectory direction in CRC1. F Changes in pathway activity of CAF subtypes along the trajectory direction in CRC1. [file 12943_2023_1876_MOESM23_ESM.tif]

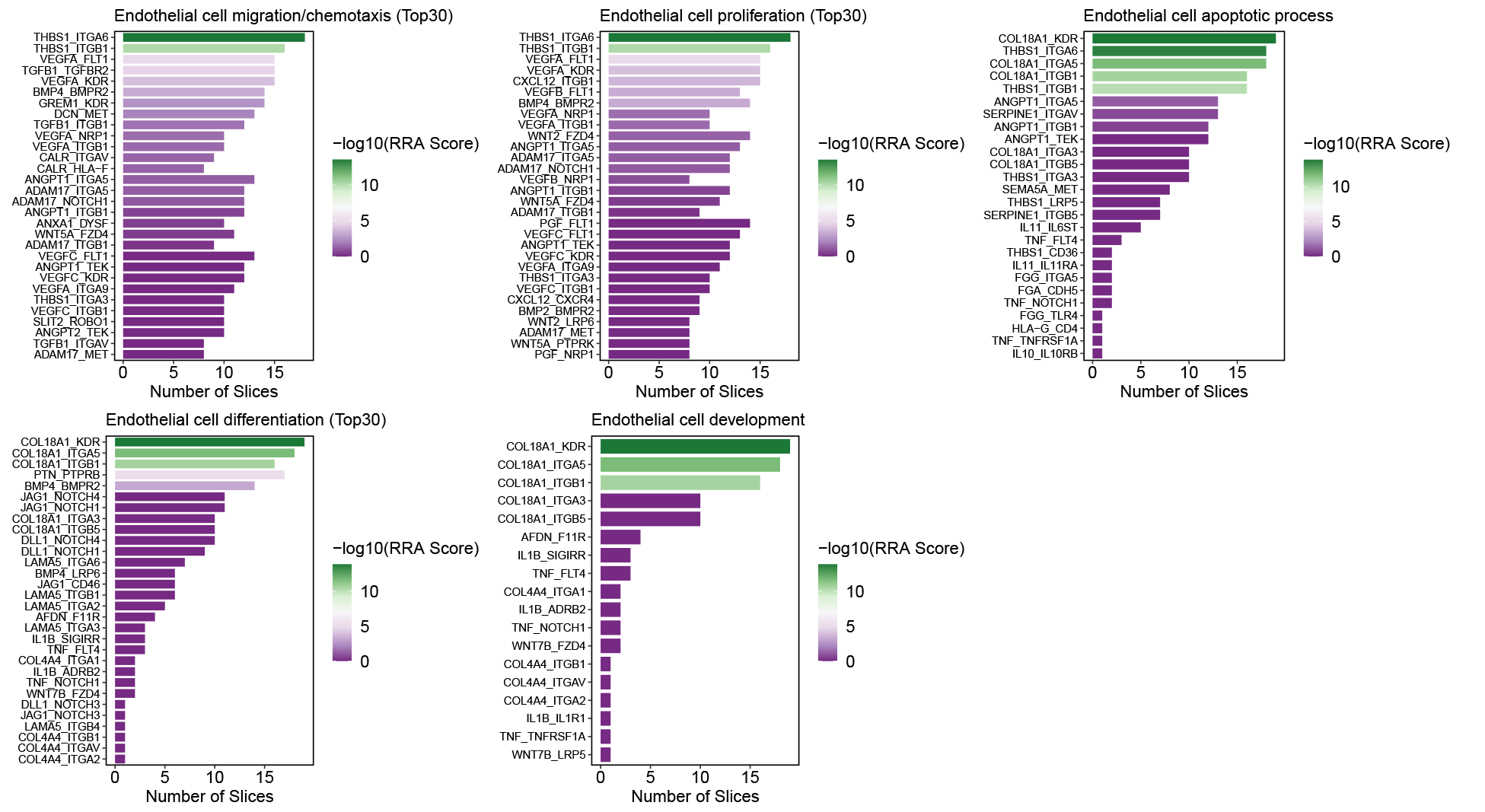

Supplement: Supplementary file 24 — Additional file 24: Figure S13. Integrated ranking of various functional LRIs based on number of LRIs from mCAFs to endothelial cells using RRA algorithm across 22 tissue slices. [file 12943_2023_1876_MOESM24_ESM.tif]

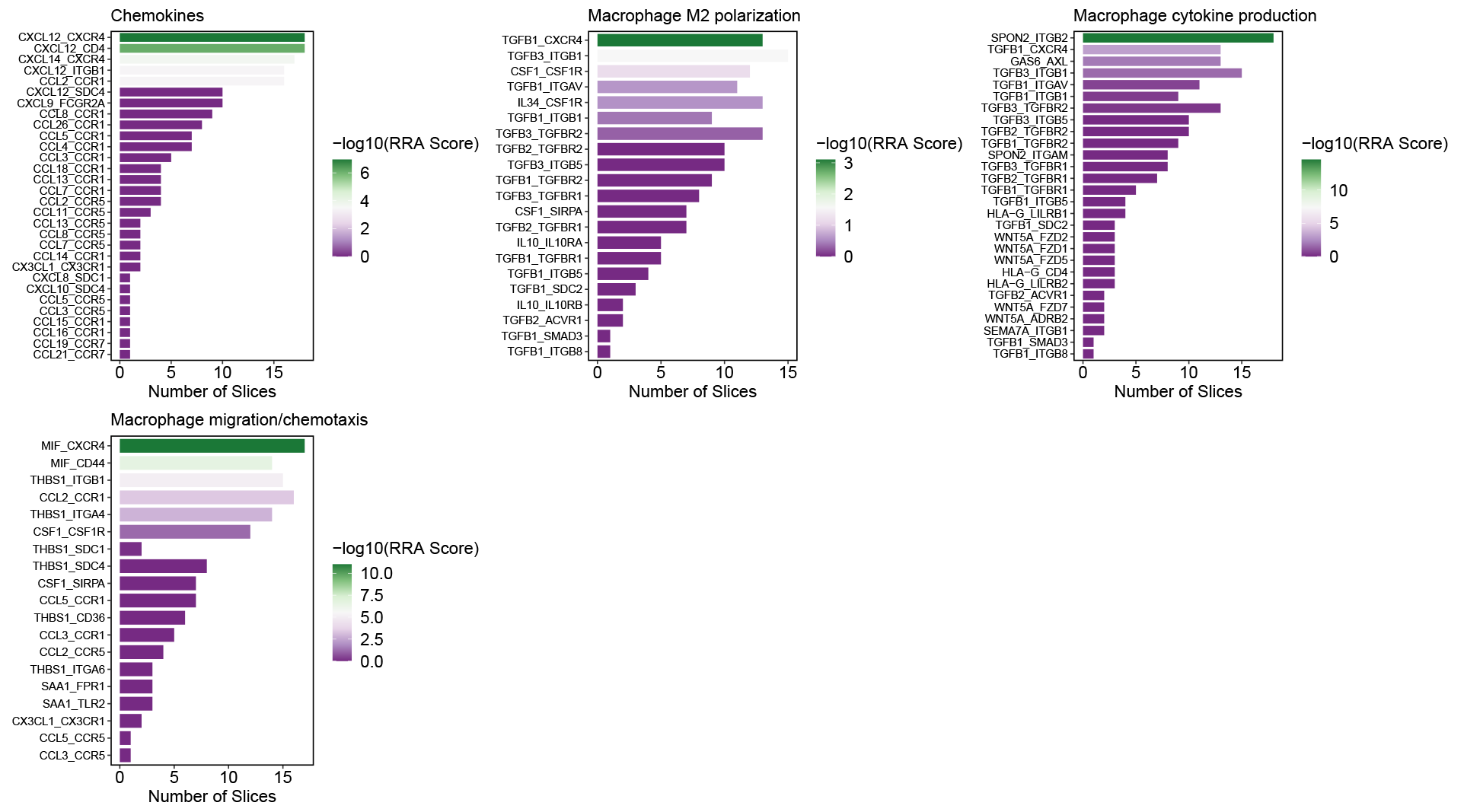

Supplement: Supplementary file 25 — Additional file 25: Figure S14. Integrated ranking of various functional LRIs based on number of LRIs from iCAFs to macrophages using RRA algorithm across 22 tissue slices. [file 12943_2023_1876_MOESM25_ESM.tif]

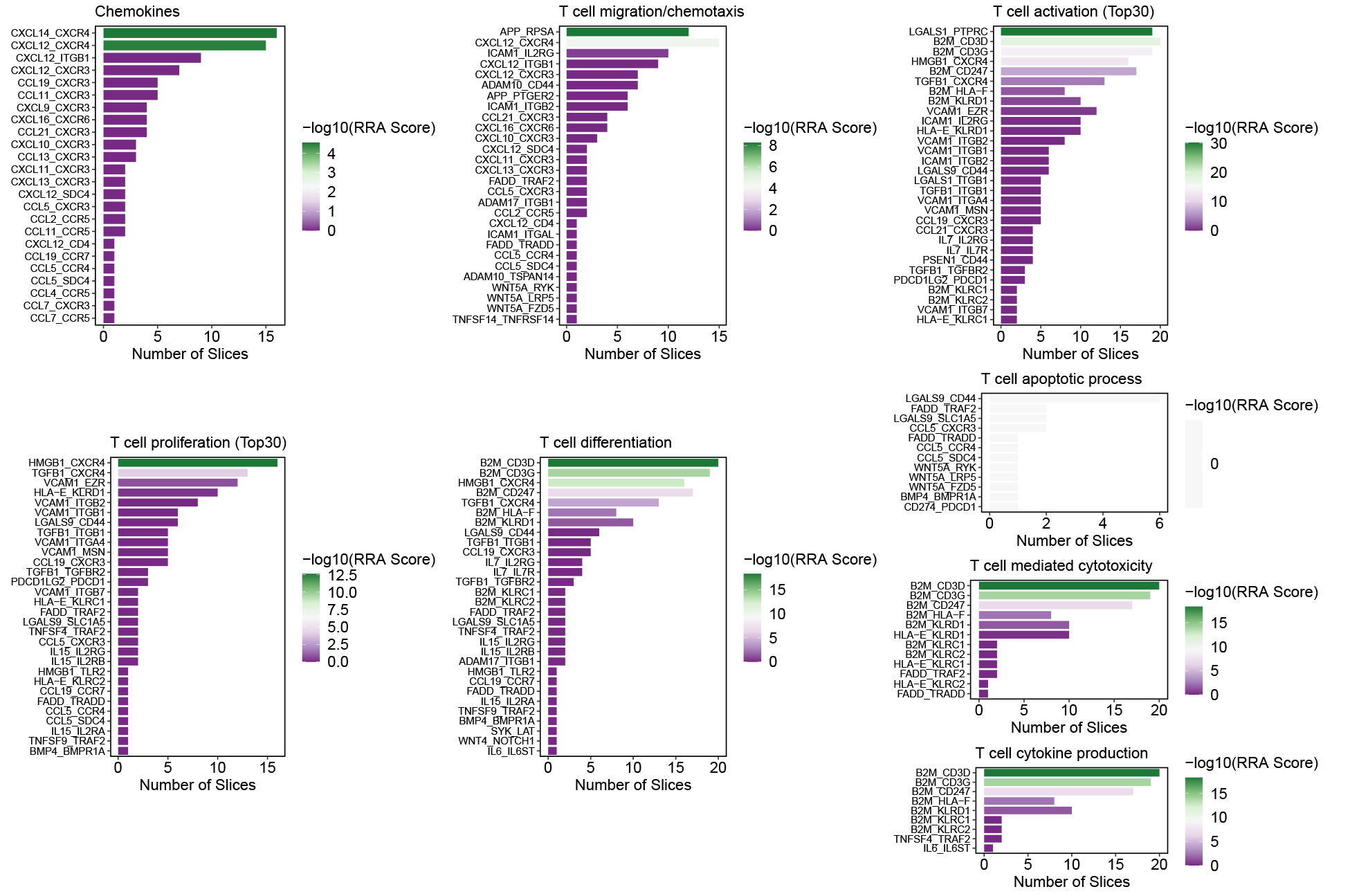

Supplement: Supplementary file 26 — Additional file 26: Figure S15. Integrated ranking of various functional LRIs based on number of LRIs from iCAFs to CD8+ T cells using RRA algorithm across 22 tissue slices. [file 12943_2023_1876_MOESM26_ESM.tif]

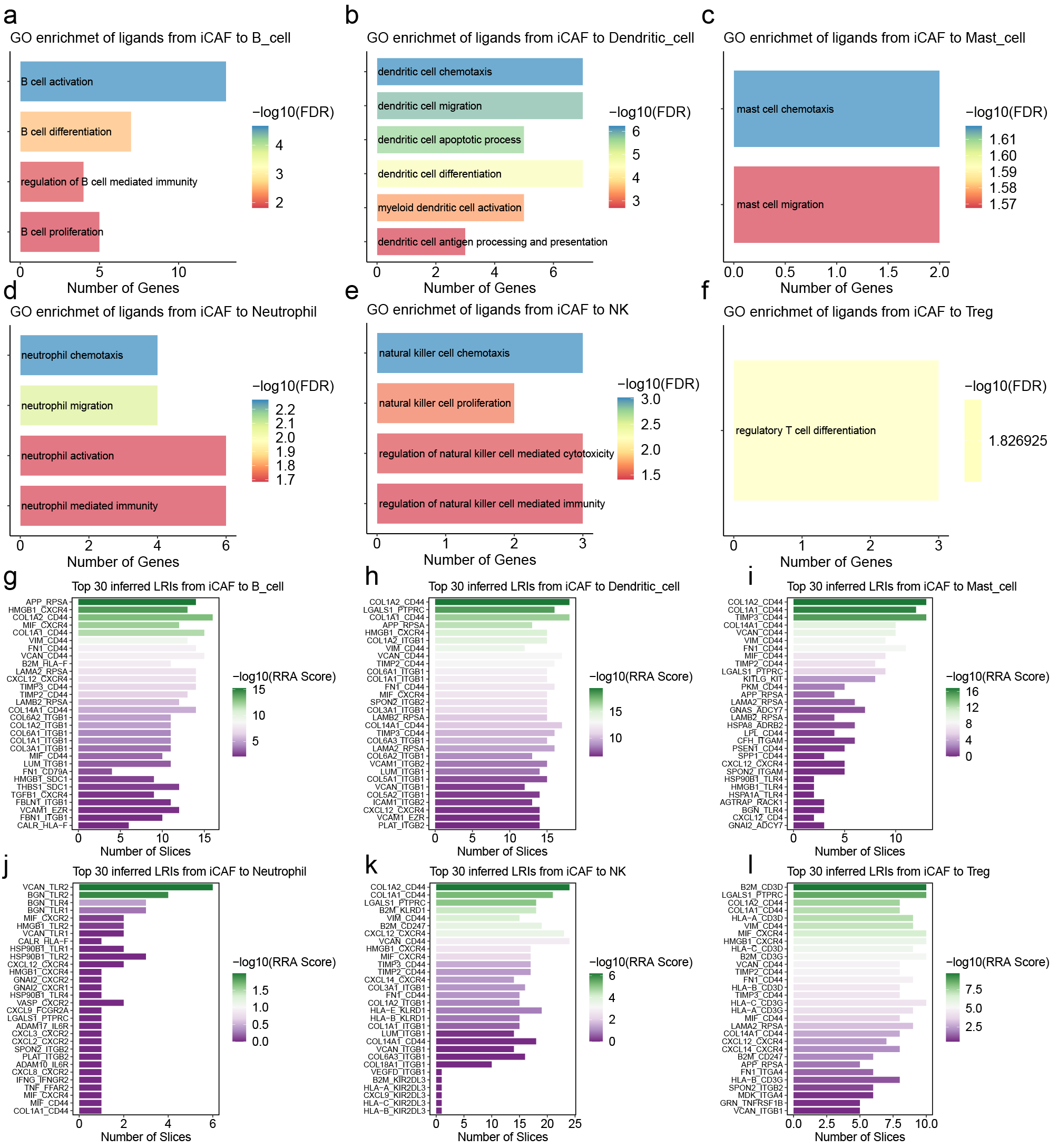

Supplement: Supplementary file 27 — Additional file 27: Figure S16. Effect of iCAFs on immune cells through paracrine signaling. A GO enrichmet of ligands from iCAFs to B cells. b GO enrichmet of ligands from iCAFs to dendritic cells. c GO enrichmet of ligands from iCAFs to mast cells. d GO enrichmet of ligands from iCAFs to neutrophils. E GO enrichmet of ligands from iCAFs to NK cells. f GO enrichmet of ligands from iCAFs to Tregs. G Integrated ranking of LRIs based on number of LRIs from iCAFs to B cells using RRA algorithm across 22 tissue slices. h Integrated ranking of LRIs based on number of LRIs from iCAFs to dendritic cells using RRA algorithm across 22 tissue slices. i Integrated ranking of LRIs based on number of LRIs from iCAFs to mast cells using RRA algorithm across 22 tissue slices. j Integrated ranking of LRIs based on number of LRIs from iCAFs to neutrophils using RRA algorithm across 22 tissue slices. k Integrated ranking of LRIs based on number of LRIs from iCAFs to NK cells using RRA algorithm across 22 tissue slices. l Integrated ranking of LRIs based on number of LRIs from iCAFs to Tregs using RRA algorithm across 22 tissue slices. [file 12943_2023_1876_MOESM27_ESM.tif]

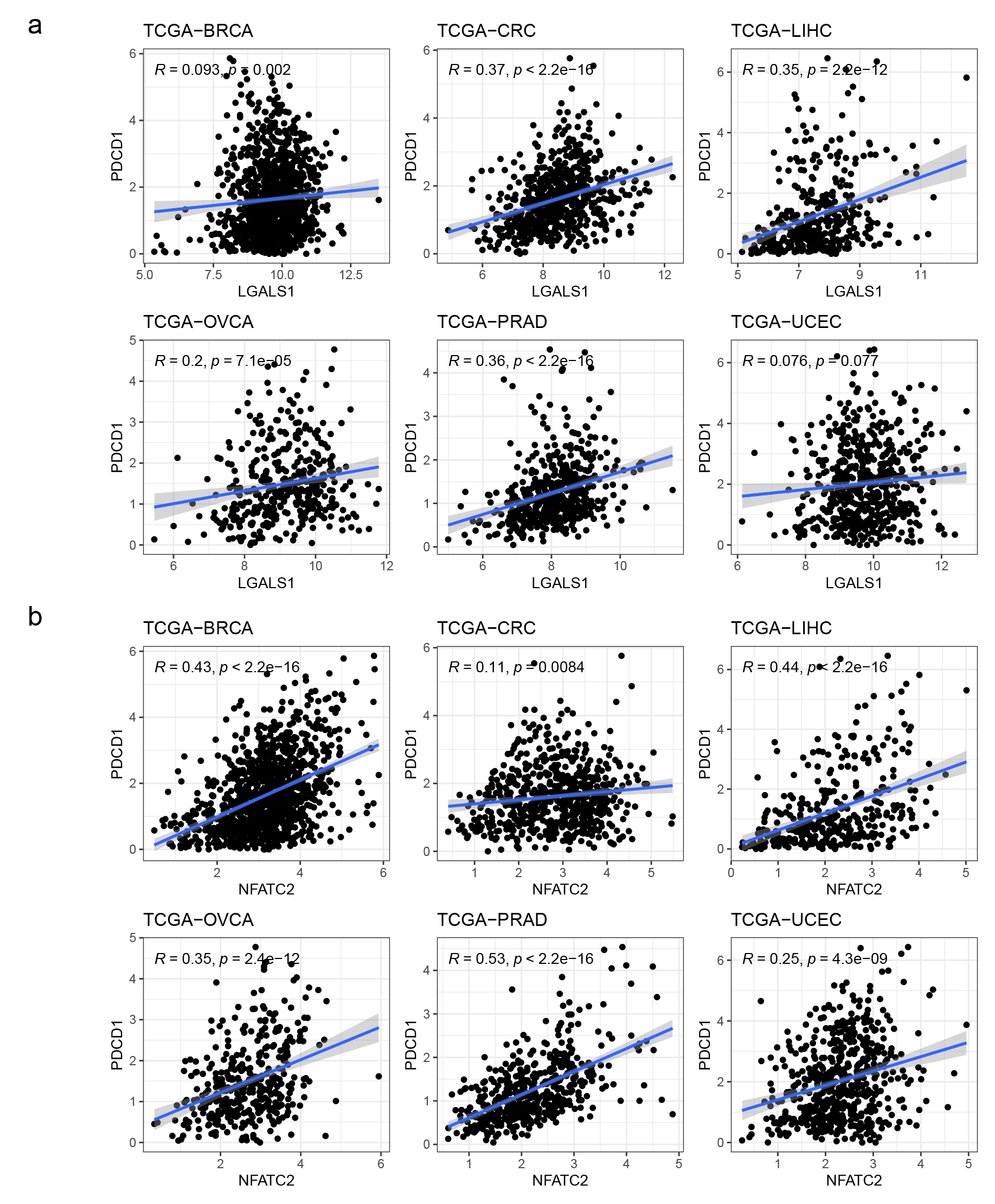

Supplement: Supplementary file 28 — Additional file 28: Figure S17. Spearman correlation analysis of LGALS1 and NFATC2 with PDCD1. A Spearman correlation analysis of LGALS1 with PDCD1. B Spearman correlation analysis of NFATC2 with PDCD1. [file 12943_2023_1876_MOESM28_ESM.tif]

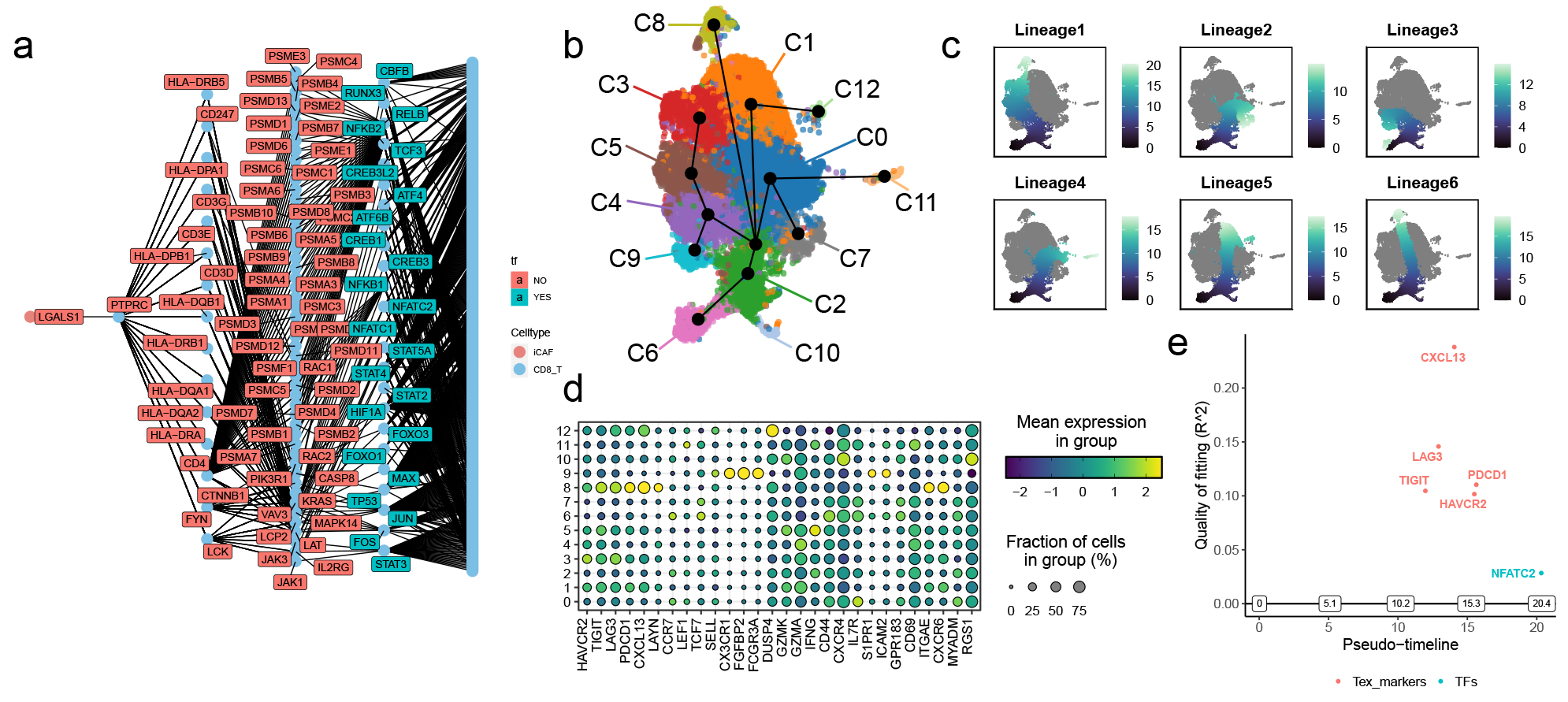

Supplement: Supplementary file 29 — Additional file 29: Figure S18. Downstream analysis of the interaction between iCAFs and CD8+ T cells. a Intracellular signaling network triggered by LGALS1- PTPRC interaction. B Slingshot trajectory analysis of the 13 clusters of CD8+ T cells. c Pseudotime of the 6 lineages of CD8+ T cells calculated by Slingshot. D Bubble heatmap showing the expression of marker genes for CD8+ T cell subtypes in pan-cancer. E GeneSwitches analysis of switching genes in Lineage 1. [file 12943_2023_1876_MOESM29_ESM.tif]

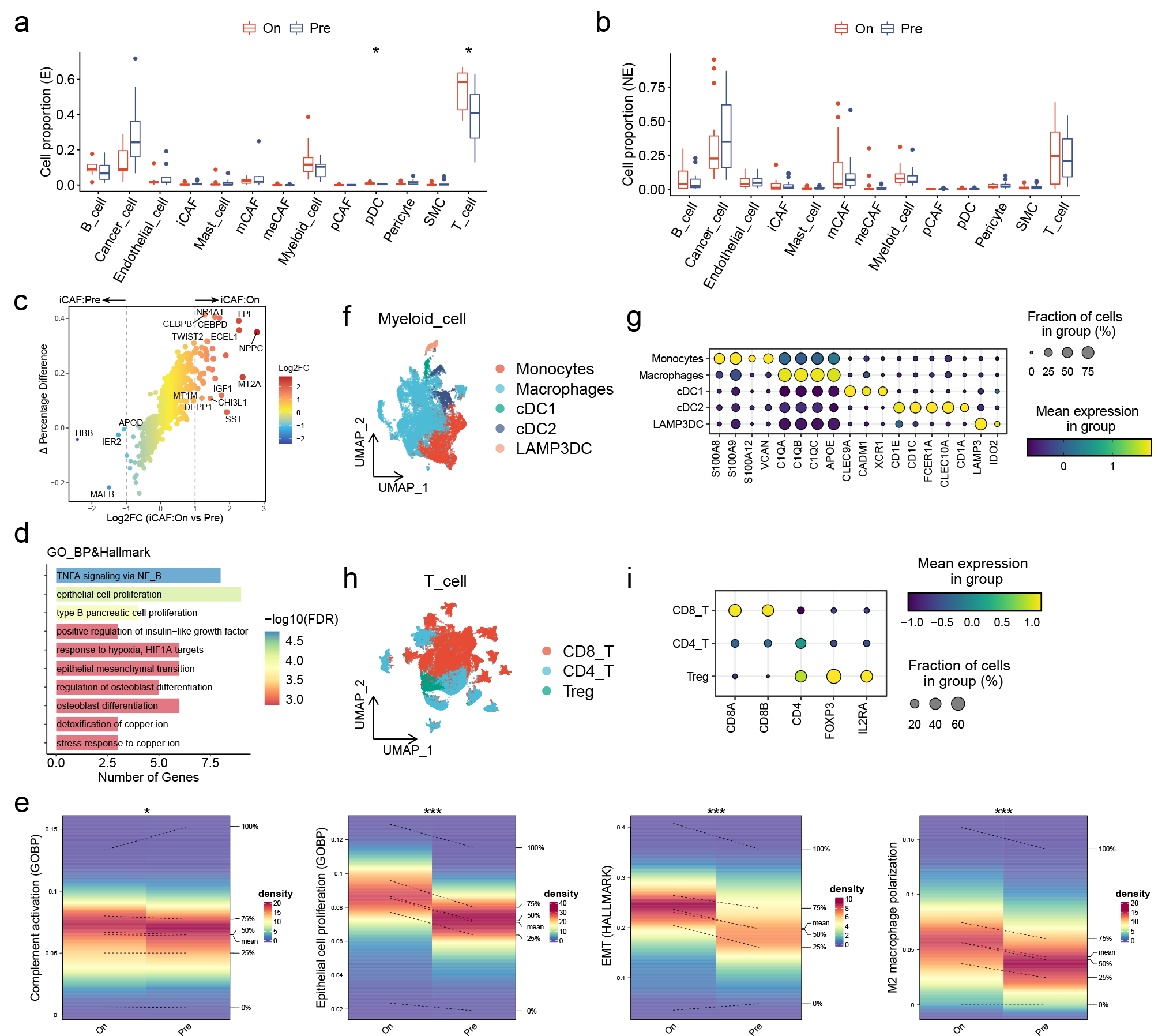

Supplement: Supplementary file 30 — Additional file 30: Figure S19. Anti-PD1 treatment influences transcriptional characteristics of iCAFs. A Boxplot showing the differences in cell proportions between patients with clonal expansion before and during anti-PD-1 treatment. Statistical analysis was performed using unpaired t-tests; *P< 0.05, **P< 0.01, ***P< 0.001. b Boxplot showing the differences in cell proportions between patients without clonal expansion before and during anti-PD-1 treatment. Statistical analysis was performed using unpaired t-tests; *P< 0.05, **P< 0.01, ***P< 0.001. c Volcano plot showing DEGs of iCAFs between pre- and on-anti-PD-1 treatment. d GO enrichmet of DEGs of iCAFs between pre- and on-anti-PD-1 treatment. e Density heatmaps showing the comparison of pathway activities of iCAFs scored by AUCell between pre- and on-anti-PD-1 treatment. Statistical analysis was performed using Wilcoxon rank-sum tests; *P< 0.05, **P< 0.01, ***P< 0.001. f UMAP plot showing the myeloid cells subpopulations in BRCA immunotherapy cohort. g Bubble heatmap showing the expression of marker genes for myeloid cells subpopulations in BRCA immunotherapy cohort. h UMAP plot showing the T cells subpopulations in BRCA immunotherapy cohort. i Bubble heatmap showing the expression of marker genes for T cells subpopulations in BRCA immunotherapy cohort. [file 12943_2023_1876_MOESM30_ESM.tif]

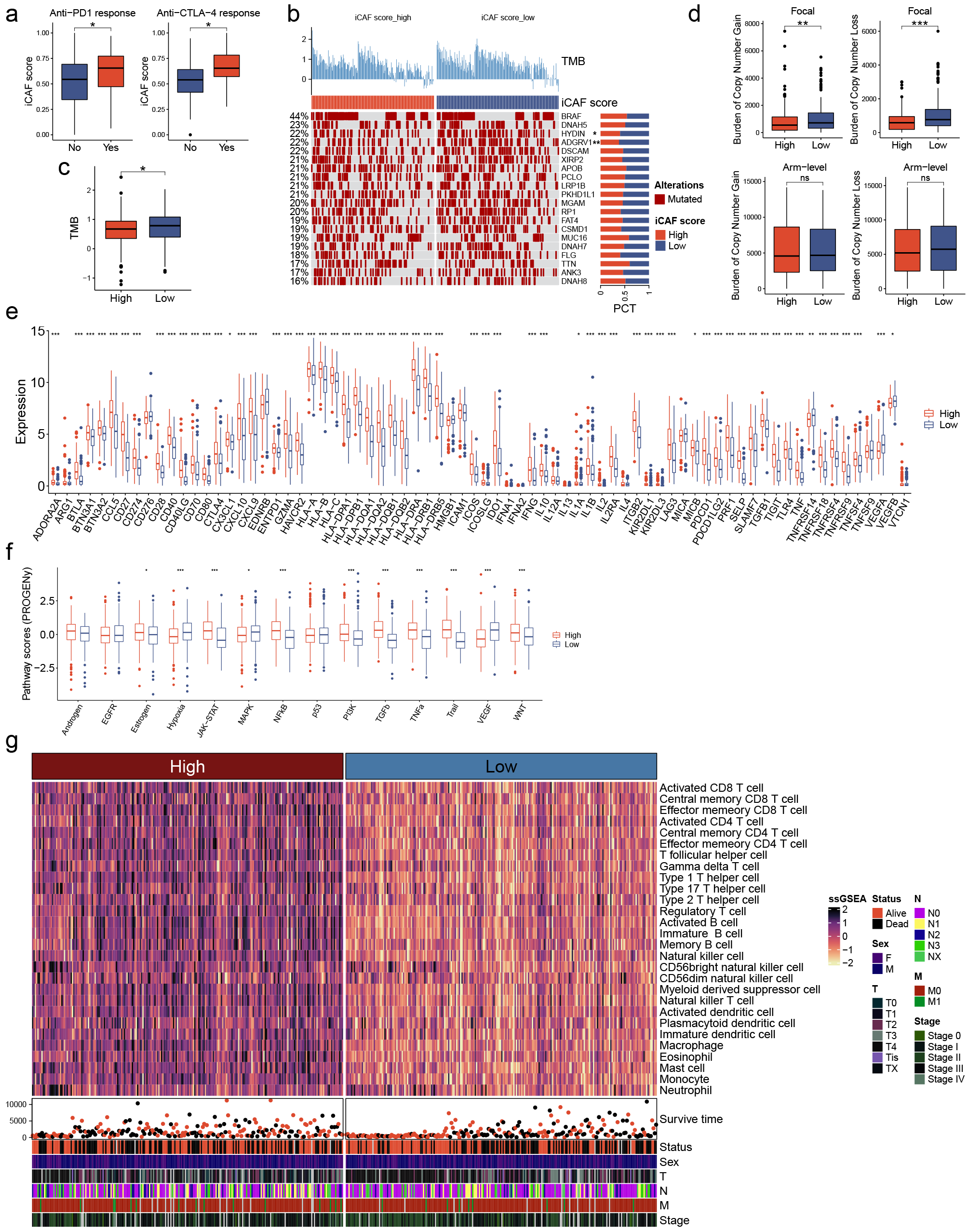

Supplement: Supplementary file 31 — Additional file 31: Figure S20. The immune and genomic landscape of melanoma patients based on high and low iCAF scores. a Boxplot showing the comparison of iCAF scores in anti−PD1 therapy responders and non-responders, as well as anti−CTLA−4 therapy responders and non-responders, among melanoma patients. Statistical analysis was performed using Wilcoxon rank-sum tests; *P< 0.05, **P< 0.01, ***P< 0.001. b Mutation landscapes of melanoma patients with high and low iCAF scores. Statistical analysis was performed using Fisher's exact test; *P< 0.05, **P< 0.01, ***P< 0.001. c Boxplot showing the comparison of TMB in melanoma patients with high and low iCAF score. Statistical analysis was performed using Wilcoxon rank-sum tests; *P< 0.05, **P< 0.01, ***P< 0.001. d Boxplot showing the comparison of copy number gain burden or copy number loss burden at arm-level or focal-level in melanoma patients with high and low iCAF score. Statistical analysis was performed using Wilcoxon rank-sum tests; *P< 0.05, **P< 0.01, ***P< 0.001. e Boxplot showing the comparison of immune modulators expression levels in melanoma patients with high and low iCAF score. Statistical analysis was performed using Wilcoxon rank-sum tests; *P< 0.05, **P< 0.01, ***P< 0.001. f Boxplot showing the comparison of pathway activities scored by PROGENy in melanoma patients with high and low iCAF score. Statistical analysis was performed using Wilcoxon rank-sum tests; *P< 0.05, **P< 0.01, ***P< 0.001. g Heatmap showing immune cell infiltration levels in melanoma patients based on high and low iCAF scores. [file 12943_2023_1876_MOESM31_ESM.tif]
